# Temporal dynamics of CBT emotion regulation training in patients with major depression using ecological momentary assessment

**DOI:** 10.1038/s41598-025-16344-3

**Published:** 2025-08-30

**Authors:** Hendrik Laicher, Isabell Int-Veen, Leonie Woloszyn, Ariane Wiegand, Agnes Kroczek, Daniel Sippel, Glenn Lawyer, Florian Torka, Vanessa Nieratschker, Julian Rubel, Andreas Fallgatter, Ann-Christine Ehlis, David Rosenbaum

**Affiliations:** 1 Department of Psychiatry and Psychotherapy, University Hospital of Tuebingen, Calwerstraße 14, 72076 Tuebingen, Germany; 2 German Center for Mental Health (Deutsches Zentrum für Psychische Gesundheit DZPG), Tuebingen, Germany; 3 Max Planck Fellow Group Precision Psychiatry, Max Planck Institute of Psychiatry, Munich, Germany; 4 Machine Learning Solutions, Luxembourg, Luxembourg; 5 Clinical Psychology & Psychotherapy of Adulthood, School of Human Sciences, Osnabrueck University, Osnabrueck, Germany; 6 LEAD Graduate School & Research Network, University of Tuebingen, Tuebingen, Germany

**Keywords:** Ecological momentary assessment (EMA), Major depressive disorder (MDD), Rumination, Repetitive negative thinking, Emotion regulation (ER), Time-lagged segment analyses, Randomized controlled trials, Depression

## Abstract

Rumination is known to have a negative impact on both the process of psychotherapy as well as the development of depressive symptoms and even the probability of relapses in Major Depressive Disorder (MDD). Therefore, treatment approaches focusing on strategies to overcome rumination have been developed over the last decades, e.g., by improving emotion regulation. However, little is yet known about the specific mechanisms of action of psychotherapy between sessions, and in particular analyses of time-dependent changes are scarce. Therefore, we used ecological momentary assessment (EMA) to collect data related to psychotherapy and well-being twice a day during a mindfulness-based emotion regulation training (MBERT). To this end, 40 patients suffering from MDD participated in a block-randomized controlled trial, consisting of an eight-session MBERT and an active wait-list control. Results give further support to the effectiveness of mindfulness-based therapy in the treatment of MDD by showing its effects in ecologically valid circumstances: Throughout MBERT, subjective stress and rumination decreased, whereas self-efficacy, self-kindness, mindful distancing and sleep quality increased. While internal causes and social interactions initially had a strong impact on subjective stress and rumination, patients were less vulnerable to such triggers after receiving MBERT. Regarding the temporal dynamics of these changes, self-efficacy, self-kindness and mindful distancing reciprocally enhanced each other, followed by a reduction in stress later on. The robustness and implications of the results are discussed.

**Supplementary Information:**

The online version contains supplementary material available at 10.1038/s41598-025-16344-3.

## Introduction

Major Depressive Disorder (MDD), one of the most prevalent mental disorders^1^, not only poses a high burden on affected patients but also on society in general^2^ and is therefore considered a leading contributor to the global burden of disease^ 3,4^. Even though not included in the official diagnostic criteria, one common key characteristic of MDD is rumination^5–8^. Originally, Nolen-Hoeksema defined rumination as “behavior and thoughts that [repetitively] focus one’s attention on one’s depressive symptoms and on the [causes, meanings, consequences and] implications of these symptoms”^ 9^, p. 569). As a perseverative, negative, self-referential, abstract thinking style with little or no goal and change orientation^10^, rumination nowadays is seen as a form of repetitive negative thinking, such as worry^11^. In the context of MDD, rumination has been found to be associated with prolonged and more severe negative affect and depressive symptoms, impaired executive functions like concentration, problem solving, and inhibition of instrumental behavior^12^ as well as reduced treatment success, delayed recovery after cognitive-behavioral therapy (CBT) and higher risk for relapses and suicidality^13–21^. Moreover, rumination leads to increased experience of stress^12^, and on the other hand has been found to be increasingly elicited by the experience of (social) stress itself^22–27^. Accordingly, rumination not only seems to be a trait-like process but can also be categorized as a (maladaptive) emotion regulation strategy (ERS)^20,28,29^. Recent findings about the associations of rumination with reduced emotional wellbeing, emotion dysregulation^30,31^ and higher physiological stress parameters^32^ lend support to this hypothesis. In the Self-Regulatory Executive Function (S-REF) model^33^, rumination is conceptualized as a part of a maladaptive coping mode, driven by dysfunctional metacognitive beliefs and sustained attentional focus on negative self-referent content. Similarly, the attentional scope model^34^ posits that negative mood narrows attentional breadth, thereby increasing the likelihood of ruminative thinking. These theories highlight the importance of enhancing attentional flexibility, metacognitive distancing, and acceptance of negative affect in reducing rumination. While rumination is widely conceptualized as a form of maladaptive emotion regulation, we would like to open the discussion that it may instead represent the result of absent or failed regulation – or even a cognitive-affective component of the emotional response itself – rather than an intentional regulatory strategy (see supplementary material).

Independent of the conceptualization of rumination, it seems important to take this phenomenon into account in the treatment of MDD, both by directly treating it and by considering it in the evaluation of therapeutic processes and treatment effects. Based on the given discussion, interventions that foster adaptive emotion regulation – such as mindfulness, acceptance or reappraisal – may reduce not only the experience of negative emotions, but also the tendency to ruminate. Therefore, we conducted a randomized controlled treatment study for MDD, investigating the efficacy of a mindfulness-based emotion regulation training (MBERT) focusing on burdening topics that patients were ruminating about. In the therapeutic approach, we followed the Emotion Regulation Therapy (ERT) established by Mennin et al.^35,36^, which has been shown to reduce self-referential processes such as rumination and worry in patients with MDD and generalized anxiety disorder^35–37^. While ERT in these studies consisted of 16 to 20 sessions and taught patients ERS through attentional control and metacognitive techniques, we used a shorter intervention comprising eight training sessions. In doing so, we applied common CBT strategies and supplemented them with mindfulness-based techniques, as is typical for the “third wave CBT” (e.g.,^38^). More precisely, patients first received a psycho-educative session on the relationship between negative emotions and rumination, before practicing a four-step strategy for dealing with negative emotions, consisting of (1) focusing on, (2) accepting, (3) reinterpreting (i.e., cognitive reappraisal), and (4) distancing from the primary burdening emotion. This training was conducted over eight sessions and under psychotherapeutic guidance. Patients were randomly assigned to either MBERT or a treatment-as-usual (TAU) control condition, which allowed patients to continue or modify their ongoing psychotherapeutic or pharmacological treatments. The study followed a crossover design, where participants initially assigned to MBERT switched to TAU and vice versa after the first treatment phase. Each phase lasted approximately four to five weeks, with a total study duration of ten weeks. Given the multifaceted nature of the study design and data collection, different aspects of the data are reported in separate publications. The present manuscript focuses specifically on the ecological momentary assessment (EMA) data collected throughout the intervention period. Previously published analyses of in-situ as well as pre-post measures revealed significant reductions in rumination and in depressive symptoms on both neural and behavioral levels, indicating that treating rumination directly is effective in treating MDD, even within a short eight-session intervention^39^. Moreover, MBERT was significantly more effective than an active wait-list control group receiving its individual TAU, i.e., patients were allowed to begin, continue or stop any psychotherapeutic or pharmacological treatment during study participation. Additionally, with the intention of tracking the effects of the treatment in everyday life and under non-laboratory conditions, we collected data about daily stress levels, stressful life events, rumination, self-efficacy, self-compassion, mindfulness and sleep quality during the study participation twice per day using EMA. These results will be reported in the present article. The used measures were chosen because they are well-suited to represent mechanisms of MBERT, and “third wave CBT” in particular, namely enhanced self-compassion^40,41^ and self-efficacy^42,43^ due to more mindfulness^44–48^: While maladaptive ERS are known to lead to higher levels of psychopathology, adaptive emotion regulation is associated with good mental health^28,49^. More precisely, previous studies found associations of self-compassion and mindfulness with reduced emotional reactivity in response to difficult everyday situations as well as with reduced negative affect in general (e.g^., 50^) while adaptive emotion regulation was significantly associated with enhanced self-efficacy (e.g.,^51^), self-compassion (e.g.,^52^) and mindfulness (e.g.,^53^). Therefore, by learning and using the MBERT strategy, patients should deal with burdening topics in a more adaptive and mindful way, which should enhance their mindfulness, self-efficacy and self-compassion even in daily life. Additionally, in line with the given discussion of rumination as a possible result of absent or ineffective emotion regulation (see supplementary material), the conscious use of the MBERT strategy should help patients regulating their burdening emotions and would therefore lead to higher self-compassion and self-efficacy (e.g., ^40–43,51,52^), whereas absent emotion regulation would have opposite effects. Furthermore, sleep quality was assessed, as it is known to be affected in MDD^54–56^ and therefore can be used as another measure of changes in the symptomatology (e.g., ^57^) and, on the other hand, psychotherapy is known to be more effective when sleep quality is good^58^.

EMA is a very promising approach to investigate processes in an ecologically valid manner without retrospective biases and therefore has already been successfully used in multiple studies, even such ones emphasizing the relationship between depressive symptoms, rumination and stress (e.g.,^59,60^), showing that stressful life events lead to higher rumination, especially in patients suffering from MDD^61^. Rosenbaum et al. (2022) further investigated the associations of different ERS with coping‑efficacy, rumination and stress in 21 depressed patients and 23 healthy controls using EMA^51^. The results of this study demonstrate that ruminative thinking increased in response to life stress, especially social interactions, but participants were able to significantly increase their self-efficacy and to reduce rumination and stress later on by using (cognitive) ERS. In the present study we did not explicitly ask the patients to use ERS in response to stressful life events, but instead collected descriptive data on daily stress and the extent of rumination, self-efficacy, self-compassion and mindfulness, in order to measure the effects of MBERT outside of the sessions under daily-life circumstances and to draw conclusions about the temporal dynamics of these effects. Regarding the latter point, there is a substantial body of research and literature that attempts to identify mediators for the effectiveness of psychotherapy, even focused on the treatment of MDD (e.g.,^62,63^). However, besides the challenges of identifying clear mediators as various studies differ in their methodology, design, and analyses ^62,64^, there are also only few studies that examine the temporal dynamics of the changes (e.g.,^65–68^) and/or try to identify models or networks that might explain how psychotherapeutic treatments work^69^. As it is known that “a successful psychotherapy continues to have an effect after a session and between sessions”^70^, p. 1062), it is of great importance to also collect and analyze data capturing such effects. For this purpose, the use of EMA is well-suited^64^. However, previous studies mostly focused on inter-session effects, capturing therapy-related thoughts and feelings, and were thus consciously and directly related to the therapy and/or specific therapeutic interventions, even if with temporal distance (e.g.,^70–73)^. In addition to this crucial research, it is also important to consider investigating general processes during psychotherapeutic treatment. This could be realized by investigating the impact of psychotherapy on further variables that might change due to the treatment, i.e., primary (e.g., self-compassion, stress) and secondary outcomes (e.g., sleep quality, changes in how far daily life triggers evoke stress), but might not always seem to be consciously related to therapy. This appears promising for gaining further insights into the general mechanisms of action of interventions of both specific and symptom-focused treatments as well as psychotherapy in general. To this end, it seems worthwhile to examine the temporal changes of such variables in patients’ daily lives while they receive psychotherapy. Accordingly, Snippe et al. (2024) did this by investigating the within-person temporal order of emotional, cognitive, and behavioral improvements during psychotherapy in patients suffering from MDD^74^. Using EMA data, they found cognitive and emotional gains to temporally co-occur, while gains in behavior seemed to follow those emotional gains. While these results are very promising to better understand psychotherapeutic processes on those three levels (emotional, cognitive, and behavioral), in our study we investigated those processes on the cognitive level in more detail. Furthermore, in contrast to Snippe et al. (2024), where psychotherapy was not provided as part of the study^74^, we controlled for possible differences in psychotherapeutic approaches by applying MBERT as part of the study procedure. Thus, we further were able to draw more precise conclusions about the concrete impact of the therapeutic approach on the observed changes.

Based on the described background, our hypotheses were as follows: First, we assumed that MBERT effectively contributes to a reduction in psychological distress and an improvement in mental health: While we have already demonstrated that MBERT significantly reduced depressive symptoms and rumination and significantly increased self-efficacy and self-compassion in both pre-post comparisons and in situ^39^, we now hypothesized that this development would also be reflected in patients’ daily lives by reduced subjective stress and rumination, as well as increased self-efficacy, self-compassion, and mindful distancing as well as improved sleep quality. Furthermore, we assumed that these effects would significantly differ between the groups (treatment vs. TAU), in the sense that stronger and faster changes would be observed in the treatment group compared to TAU. Concerning the temporal dynamics of these changes, we performed an exploratory analysis to investigate possible segment effects, by examining the influence of each variable on every other variable. Furthermore, we assumed that the patients’ handling of daily life stress-triggering events would change through learning the MBERT strategy, in the sense that such events would trigger less subjective stress and rumination, as patients would have learned a more constructive way of dealing with them. Finally, based on our preliminary studies^26,51^, we hypothesized that patients would generally experience and report fewer stress-triggering events throughout their study participation with a stronger reduction during treatment vs. TAU.

## Results

### Participant descriptives and demographics

In total, 4925 complete data entries from 40 patients could be included in the data analysis with a mean of *M* = 123.125 (*SD* = 26.452) data entries per patient. This corresponds to 72.67 % of all assessment points for all patients. The duration of EMA ranged from 43 days to 113 days across all patients with a mean duration of *M* = 76.475 (*SD* = 14.058) days. There was no significant difference in the amount of data entries between the randomized groups (group 1 receiving MBERT before switching to TAU; group 2 having the TAU phase before receiving MBERT) (*t*(38) = -0.806, *p* > .05, *d* = 0.26), however, patients answered the EMA significantly more frequently during the MBERT phase (*M* = 69.000 data entries; *SD* = 21.703 data entries) compared to the TAU phase (*M* = 54.125 data entries; *SD* = 17.625 data entries) (*t*(75) = -3.365, *p* < .01, *d* = 0.75).

### Multilevel factor analysis of the used items

First, we aimed to explore the factorial structure of the items used in the assessment of rumination, self-efficacy, self-kindness and mindful distancing. To this end, we performed an exploratory multilevel factor analysis with all 15 items (i.e., even those taken from the already validated questionnaires RRS, PCQ and SCS-D) with oblique rotation (oblimin) in MPlus. The results confirmed that a five-factor solution was sufficient for the data, resulting in one factor for self-efficacy, self-kindness and mindful distancing, consisting of the corresponding items. Concerning the six rumination items, those taken from the RRS and those taken from the PCQ loaded on two separate factors, underlining the different aspects of rumination they assess: While the PCQ assesses the uncontrollability and the repetitiveness of ruminative thoughts, the RRS focuses on the negative and self-related content of those thoughts (i.e., own problems and failures). However, as both aspects are part of the general phenomenon of rumination^10^, all six items can be seen as fitting to it. Furthermore, given the strong correlation between these two factors (*r* = .506, *p* < .05), rumination was treated as a single variable in all further analyses, incorporating all six items. Taken together, the factors rumination, self-efficacy, self-kindness and mindful distancing could be treated as different variables. For the results of the exploratory multilevel factor analysis as well as Cronbach’s alpha of the questionnaire scales, see supplementary material Table S4 and Table S5.

### Sample characteristics: correlations, baseline, halftime and endpoint values

We examined repeated measures correlations^75,76^ between all dependent variables. As expected, rumination and stress were positively correlated; all other dependent variables (self-efficacy, self-kindness, mindful distancing, sleep quality) were positively correlated as well. Furthermore, correlations between rumination or stress with any of the other dependent variables were negative. All correlations reached significance (see Table 1).

**Table 1:** Repeated measures correlations.

	Stress	Self-Efficacy	Self-Kindness	Mindful Distancing	Sleep Quality
Rumination	0.493***	− 0.614***	− 0.509***	− 0.581***	− 0.228***
Stress		− 0.438***	− 0.297***	− 0.361***	− 0.247***
Self-Efficacy			0.574***	0.594***	0.243***
Self-Kindness				0.606***	0.196***
Mindful Distancing					0.217***

***p < .001.

The performed rmMANOVA revealed a significant interaction effect for time and group (*Wilks λ* = 0.484, *F*(12,142) = 5.182, *p* < .001, *η*_*p*_*²* = 0.31), composed of significant interactions for rumination (*F*(2,76) = 6.196, *p*_*corr*_ < 0.05, *η*_*p*_*²* = 0.14), self-efficacy (*F*(2,76) = 7.550, *p*_*corr*_ < 0.05, *η*_*p*_*²* = 0.17), self-kindness (*F*(2,76) = 32.065, *p*_*corr*_ < 0.05, *η*_*p*_*²* = 0.46), and mindful distancing (*F*(2,76) = 16.089, *p*_*corr*_ < 0.05, *η*_*p*_*²* = 0.30), but not for stress (*F*(2,76) = 0.478, *p*_*corr*_ > 0.05, *η*_*p*_*²* = 0.01) or sleep quality (*F*(2,76) = 1.707, *p*_*corr*_ > 0.05, *η*_*p*_*²* = 0.04). All of the significant interactions were characterized by a quadratic relationship (rumination: *F*(1,38) = 12.677, *p* < .01, *η*_*p*_*²* = 0.25; self-efficacy: *F*(1,38) = 15.405, *p* < .001, *η*_*p*_*²* = 0.29; self-kindness: *F*(1,38) = 66.903, *p* < .001, *η*_*p*_*²* = 0.64; mindful distancing: *F*(1,38) = 30.263, *p* < .001, *η*_*p*_*²* = 0.44), indicating expected time-delayed u-shaped changes between the groups (see Figs. 2a-f). However, the time × group interactions for self-kindness and mindful distancing were also characterized by linear polynomial contrasts (self-kindness: *F*(1,38) = 4.446, *p* < .05, *η*_*p*_*²* = 0.11; mindful distancing: *F*(1,38) = 5.237, *p* < .05, *η*_*p*_*²* = 0.12). Furthermore, the main effect of time reached significance (*Wilks λ* = 0.232, *F*(12,142) = 12.747, *p* < .001, *η*_*p*_*²* = 0.52), composed of significant main effects for all dependent variables but sleep quality (rumination: *F*(2,76) = 34.409, *p*_*corr*_ < 0.01, *η*_*p*_*²* = 0.48; stress: *F*(2,76) = 16.644, *p*_*corr*_ < 0.05, *η*_*p*_*²* = 0.31; self-efficacy: *F*(2,76) = 41.258, *p*_*corr*_ < 0.05, *η*_*p*_*²* = 0.52; self-kindness: *F*(2,76) = 95.831, *p*_*corr*_ < 0.05, *η*_*p*_*²* = 0.72; mindful distancing: *F*(2,76) = 75.694, *p*_*corr*_ < 0.05, *η*_*p*_*²* = 0.67; sleep quality: *F*(2,76) = 2.332, *p*_*corr*_ > 0.05, *η*_*p*_*²* = 0.06). All significant main effects were characterized by a linear relationship (rumination: *F*(1,38) = 64.189, *p* < .001, *η*_*p*_*²* = 0.63; stress: *F*(1,38) = 30.239, *p* < .001, *η*_*p*_*²* = 0.44; self-efficacy: *F*(1,38) = 68.856, *p* < .001, *η*_*p*_*²* = 0.64; self-kindness: *F*(1,38) = 171.445, *p* < .001, *η*_*p*_*²* = 0.82; mindful distancing: *F*(1,38) = 131.873, *p* < .001, *η*_*p*_*²* = 0.78).

**Fig. 1:**
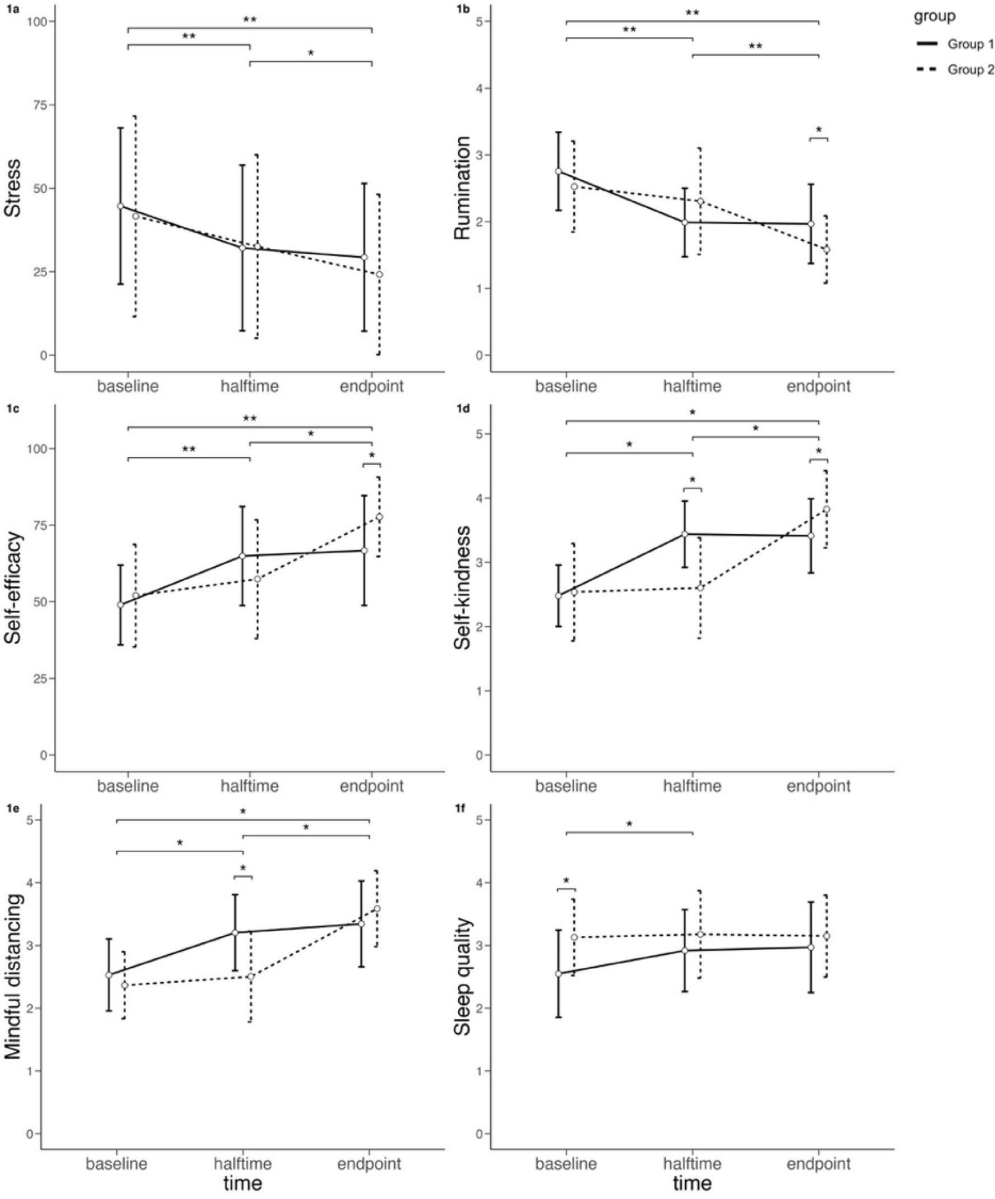
Changes in (**a**) subjective stress, (**b**) rumination, (**c**) self-efficacy, (**d**) self-kindness, (**e**) mindful distancing, and (**f**) sleep quality, reported via EMA in the course of the study participation, differentiated by group. Group 1 received MBERT first before continuing with TAU, whereas group 2 received the MBERT after a TAU phase. Small brackets indicate significant group differences.

Post-hoc comparisons within the groups revealed significant reductions in rumination and stress as well as significant increases in self-efficacy, self-kindness and mindful distancing between all time points, i.e., from baseline to halftime, from halftime to endpoint, and from baseline to endpoint. Furthermore, sleep quality significantly improved from baseline to halftime. However, the between-groups *t*-tests at halftime only reached significance for self-kindness and mindful distancing, indicating stronger increases in these two variables due to MBERT compared to TAU during the first half of the study. Note that groups didn’t differ in self-kindness at baseline even though there was a significant group difference in the initial SCS-D score at the beginning of the study (see Methods section) and items used in the assessment of self-kindness were taken from the SCS-D. This might be due to the reduced number of items assessed via EMA, and/or the initial difference might have diminished within the first days of EMA assessment. Interestingly, groups differed significantly in sleep quality at baseline, but this difference was no longer found at halftime or endpoint, after sleep quality significantly increased from baseline to halftime over both groups. Note, however, that these effects need to be interpreted with caution as neither the interaction nor the main effect for sleep quality reached significance. Regarding the endpoint values, groups differed significantly in rumination, self-efficacy and self-kindness with higher rumination as well as lower self-efficacy and self-kindness in group 1 compared to group 2 (see Figs. 1a-f). For detailed results of the rmMANOVA and all post-hoc tests see Table S6 in the supplementary material.

### Growth models

Before analyzing the temporal dynamics of the found changes, we first calculated a basic model, analyzing the impact of study phase (MBERT vs. TAU) and time (with each data entry representing one time stamp) on each dependent variable:

$${\text{Model 1}}:{\text{ Dependent variable}}\,\sim \,{\text{study phase }}*{\text{ time}}$$

Time, study phase and their interaction were included as a fixed effect. Additionally, random intercepts and random slopes for study phase were modeled at the subject level to account for individual variability in the effect of study phase. A full model specification as well as the R-syntax of this model are to be found in the supplementary material. Note that for each dependent variable we calculated this basic model with time as an uncorrected, squared as well as logarithmic variable as predictor and compared these for the best fit. For rumination, the model with the uncorrected time variable best fitted the data, whereas for all other dependent variables it was the model with the logarithmic time variable. The time variable with the best fit was further used in Model 1.

**Fig. 2:**
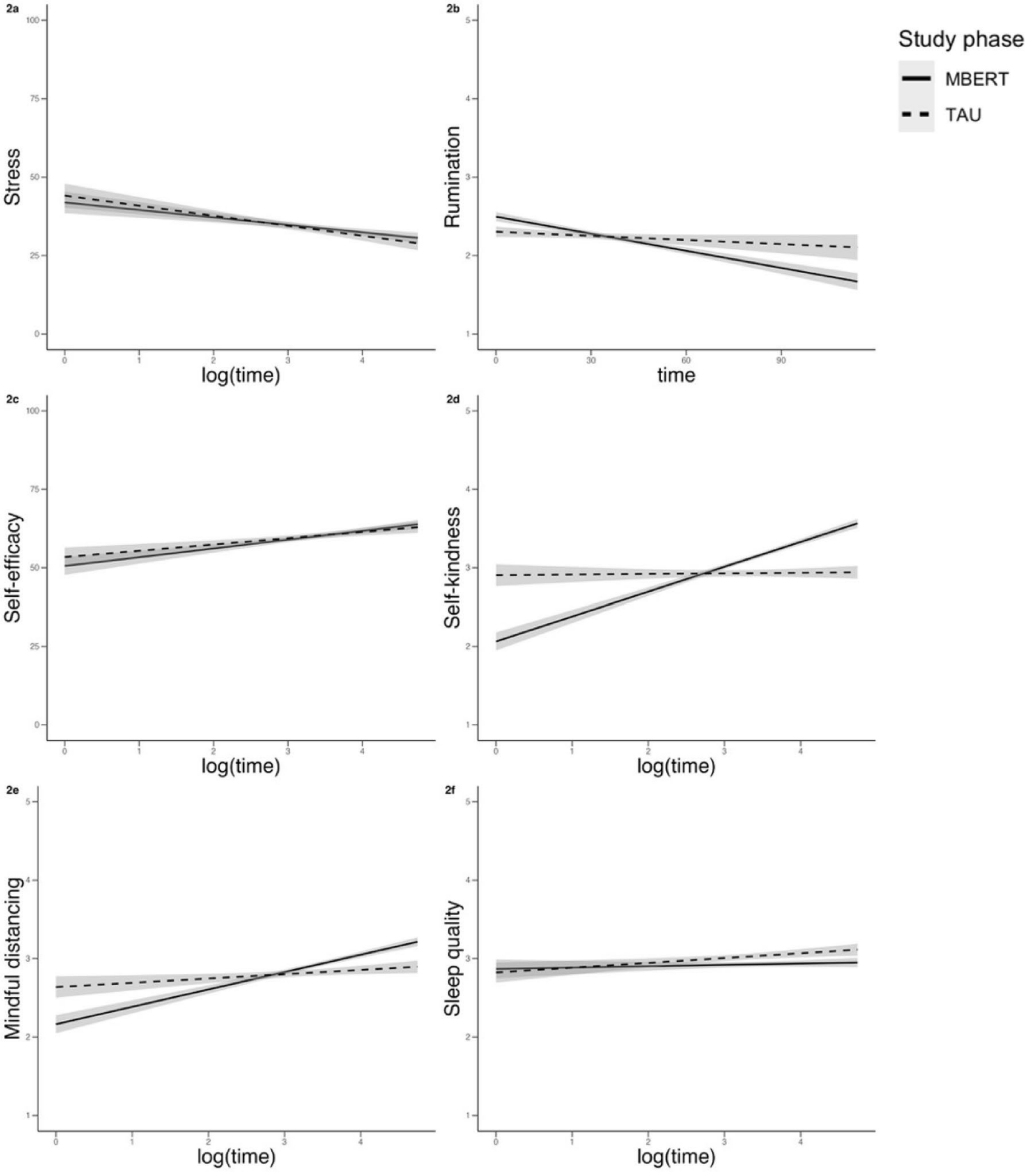
Changes in (**a**) subjective stress, (**b**) rumination, (**c**) self-efficacy, (**d**) self-kindness, (**e**) mindful distancing, and (**f**) sleep quality, reported via EMA in the course of the study participation, differentiated by study phase (MBERT vs. TAU). The shaded area displays the 95 % confidence interval.

Results of Model 1 yielded a significant study phase × time interaction in all dependent variables but stress, with rumination decreasing significantly more strongly over time during MBERT than TAU, whereas self-efficacy, self-kindness, mindful distancing and sleep quality increased significantly more strongly over time during MBERT than TAU (see Figs. 2a-f). Similar to the previously described interaction, the main effect for study phase reached significance for all dependent variables except stress and sleep quality, in the expected direction, i.e., rumination was significantly decreased whereas self-efficacy, self-kindness and mindful distancing significantly increased during MBERT vs. TAU. The results of Model 1 for each dependent variable can be found in Table 2.

Due to a violation of the assumption of normally distributed data in the cases of rumination and stress, we calculated the basic model again after log-transforming the data of those two variables, yielding the exact same results (see supplementary material Table S7).

**Table 2:** Results of the linear mixed models exploring the association between rumination, subjective stress, self-efficacy, self-kindness, mindful distancing, sleep quality and study phase and time, respectively. AIC = Akaike Information Criterion; BIC = Bayesian Information Criterion; R^2^ = variance explained by the fixed effects.

Dependent variables	Rumination	Stress	Self-Efficacy	Self-Kindness	Mindful Distancing	Sleep quality
Intercept	2.323*** (0.099)	44.580*** (2.965)	55.571*** (2.873)	2.909*** (0.128)	2.648*** (0.122)	2.966*** (0.095)
Study phase^a^	0.231* (0.098)	0.868 (2.746)	-11.805*** (2.682)	-0.969*** (0.130)	-0.614*** (0.127)	-0.172# (0.090)
Time^b^	-0.002** (0.001)	-3.304*** (0.512)	0.973** (0.359)	0.008 (0.015)	0.051** (0.016)	-0.005 (0.017)
Study phase^a^ * Time^b^	-0.008*** (0.001)	-0.595 (0.679)	4.752*** (0.475)	0.363*** (0.020)	0.228*** (0.021)	0.052* (0.022)
AIC	10592.4	44338.3	40877.2	9859.7	10129.7	10742.0
BIC	10644.4	44390.3	40929.2	9911.7	10181.7	10794.0
R^2^	0.048	0.018	0.043	0.091	0.053	0.001

^a^Coding of the study phase variable: 0 = TAU, 1 = MBERT. ^b^Note that for each dependent variable the time predictor is the one with the best fit to the data (uncorrected for rumination, logarithmic time for all other dependent variables). #*p* < .1, *p < .05, **p < .01, ***p < .001.

### Temporal dynamics of change

Next, we performed multilevel time-lagged segment analyses to investigate potential mediators and sequence effects of the changes in the outcome variables described above, using the following model:

$$\begin{gathered} {\text{Model 2}}:{\text{ Outcome variabl}}{{\text{e}}_{{\text{ t}}+{\text{1 }}|{\text{ controlled for t}}}} \sim {\text{ predictor variabl}}{{\text{e}}_{{\text{ t }}|{\text{ controlled for t}} - {\text{1}}}}*{\text{ study phase}} \hfill \\ \end{gathered}$$

with t specifying the data entry point. Random intercepts but not random slopes were included and measures were nested within patients. A full model specification as well as the R-syntax of this model are to be found in the supplementary material. Only fully consecutive data series of three assessment points in a row were considered. Therefore, assessment points were excluded, if one or both of the preceding ones were missing (i.e., patients didn’t answer EMA at these times). Accordingly, 2918 complete data series from 40 patients were included in the analysis, representing 61.26 % of the total dataset with a mean of *M* = 72.950 (*SD* = 29.080) data entries per patient. Like in the total dataset, there was no significant difference in the amount of data entries between the randomized groups (*t*(38) = -0.921, *p* > .05, *d* = 0.29). However, the previously found significant difference between the MBERT vs. the TAU phase of the study didn’t reach significance anymore (*t*(75) = -1.937, *p* > .05, *d* = 0.43). Note that sleep quality was not included in this analysis as patients answered EMA twice per day and therefore, they didn’t sleep between every two data entries.

Each dependent variable was considered both as a predictor and an outcome of all other dependent variables, accounting for every possible combination. However, for reasons of simplified presentation, results only are presented if the outcome variable was significantly predicted by the predictor variable, either as main effect or as an interaction with study phase. Results revealed that higher self-efficacy, self-kindness and mindful distancing at t led to significantly lower subjective stress at t + 1 during MBERT compared to TAU (interaction effects with study phase). However, all of these three predictor variables were themselves significantly predicted by other variables. Specifically, self-efficacy and self-kindness both were significantly lower during MBERT than during TAU at t + 1 when rumination was high at t (interaction effects with study phase). In contrast, higher mindful distancing at t generally led to higher self-efficacy and self-kindness at t + 1, respectively, independent of study phase (main effects). Furthermore, self-kindness and self-efficacy predicted each other: higher self-kindness at t generally (i.e., independently of study phase) led to higher self-efficacy at t + 1 (main effect), while higher self-efficacy at t led to higher self-kindness at t + 1 specifically during MBERT compared to TAU (interaction effect with study phase). Interestingly, we also found a main effect for stress in the prediction of self-kindness: higher stress at t was generally followed by higher self-kindness at t + 1. In addition, higher self-kindness at t led to higher mindful distancing at t + 1 across both phases (main effect). Rumination was the only variable that was not significantly predicted by any other variable. However, main effects for stress and mindful distancing reached marginal significance in predicting rumination, such that lower stress and lower mindful distancing at t preceded higher rumination at t + 1, again independent of study phase. Taken together, ruminative behavior appears to reduce self-efficacy and self-kindness later on, with a stronger reduction during MBERT compared to TAU. However, while self-efficacy, self-kindness and mindful distancing seem to enhance each other reciprocally over time regardless of study phase, this enhancement seems to reduce subjectively perceived stress – an effect that was significantly stronger during MBERT. The results of Model 2 can be found in Table 3.

**Table 3:** Results of the multilevel time-lagged segment analyses, using z-standardized data.

Outcome variable at t + 1	Stress			Self-Efficacy			Self-Kindness				Mindful Distancing
Predictor variable	Self-Efficacy	Self-Kindness	Mindful Distancing	Rumination	Self-Kindness	Mindful Distancing	Rumination	Stress	Self-Efficacy	Mindful Distancing	Self-Kindness
Intercept	26.656*** (2.731)	26.119*** (2.878)	27.391*** (0.318)	40.906*** (2.564)	23.934*** (2.095)	25.730*** (2.062)	1.775*** (0.105)	1.517*** (0.078)	1.327*** (0.084)	1.106*** (0.084)	1.160*** (0.085)
Predictor variable at t	0.068* (0.034)	0.954 (0.741)	0.367 (0.760)	0.046 (0.583)	1.695** (0.562)	1.178* (0.586)	0.031 (0.025)	0.002* (0.001)	0.0001 (0.001)	0.077** (0.026)	0.073** (0.026)
Predictor variable at t-1^a^	-0.138*** (0.025)	-2.000*** (0.567)	-1.913*** (0.556)	-2.313*** (0.392)	3.407*** (0.410)	3.045*** (0.405)	-0.104*** (0.017)	-0.002*** (0.001)	0.006*** (0.001)	0.171*** (0.018)	0.178*** (0.018)
Outcome variable at t	0.330*** (0.019)	0.321*** (0.018)	0.318*** (0.019)	0.377*** (0.020)	0.325*** (0.019)	0.358*** (0.020)	0.442*** (0.018)	0.481*** (0.017)	0.407*** (0.019)	0.379*** (0.020)	0.317*** (0.020)
Study phase^b^	4.369# (2.580)	7.679** (2.855)	5.348# (2.806)	5.625*** (1.567)	-0.942 (2.047)	0.066 (2.027)	0.284*** (0.070)	0.140*** (0.042)	-0.095 (0.080)	0.137 (0.089)	-0.037 (0.092)
Predictor variable at t * Study phase^b^	-0.079* (0.039)	-2.644** (0.900)	-2.012* (0.928)	-1.628* (0.660)	0.792 (0.646)	0.616 (0.670)	-0.078** (0.029)	-0.001 (0.001)	0.003** (0.001)	-0.011 (0.030)	0.022 (0.029)
AIC	35945.5	25953.8	25958.5	24094.6	24014.9	24062.5	5927.7	5962.8	5881.5	5850.4	5889.9
BIC	25993.3	26001.7	26006.3	24142.5	24062.7	24110.3	5975.5	6010.7	5929.3	5898.2	5937.7
R^2^	0.150	0.134	0.137	0.243	0.270	0.257	0.288	0.290	0.319	0.345	0.298

AIC = Akaike Information Criterion; BIC = Bayesian Information Criterion; R^2^ = variance explained by the fixed effects. Note that only those models with significant predictor variables (either as main effect or in an interaction with study phase) are reported here due to reasons of simplified data presentation.

^a^Note that the predictor variable at t-1 reaches higher significance than the predictor variable at t, as predictor and outcome variables are correlated and the outcome variable at t already accounts for a lot of common variance of both variables at t. ^b^Coding of the study phase variable: 0 = TAU, 1 = MBERT. #*p* < .1, *p < .05, **p < .01, ***p < .001.

### Stress and rumination decreased despite stress-inducing events

Next, we examined changes in the occurrence of the stress-evoking triggers as well as their impact on stress and rumination over the course of the study participation. After exclusion of those patients with less than 50 % of data entries in one of the three predefined phases (see Methods section), the resulting dataset consisted of 2167 data entries from 26 patients (Group 1: *n* = 11; Group 2: *n* = 15), representing 44 % of the total dataset with *M* = 54.175 (*SD* = 17.800) data entries per patient. Again, there were no significant differences in the amount of data entries between the randomized groups (*t*(36) = 0.690, *p* > .05, *d* = 0.22) nor between the study phases (*t*(72) = 0.439, *p* > .05, *d* = 0.10). Furthermore, there were also no significant differences in the amount of data entries between the three predefined phases (*F*(1,6) = 2.289, *p* > .05, *η*_*p*_*²* = 0.62). Unsurprisingly, there was a significant difference in the frequency of the different triggers, both overall as well as within each of the three phases, regardless of group assignment (see Table 4).

**Table 4:** Percentage occurrence and chi-squared tests of the different triggers overall and during the three phases, not differentiated by group.

Trigger	Percentual occurrence of the trigger			
	Overall^a^	Phase 1^b^	Phase 2^c^	Phase 3^d^
Trigger 1: No answer	38.92 %	23.15 %	42.23 %	44.78 %
Trigger 2: Social interaction	7.25 %	8.86 %	7.06 %	6.12 %
Trigger 3: Work	14.76 %	16.94 %	14.97 %	15.83 %
Trigger 4: Private obligations	7.05 %	8.75 %	6.64 %	5.58 %
Trigger 5: Daily hassles	7.92 %	10.30 %	9.46 %	6.30 %
Trigger 6: Internal causes	20.98 %	28.02 %	17.51 %	18.17 %
Trigger 7: Sleep quality	2.36 %	3.32 %	1.55 %	1.80 %
Trigger 8: Political events	0.77 %	0.66 %	0.57 %	1.44 %
*η*^*2*^-statistic	*η*^*2*^(7) = 4310.1, *p* < .001	*η*^*2*^(7) = 455.36, *p* < .001	*η*^*2*^(7) = 708.36, *p* < .001	*η*^*2*^(7) = 644.75, *p* < .001

^a^*n* = 40 patients with a total of *n* = 4925 data entries. ^b^*n* = 26 patients with a total of *n* = 903 data entries. ^c^*n* = 26 patients with a total of *n* = 708 data entries. ^d^*n* = 26 patients with a total of *n* = 556 data entries.

**Fig. 3:**
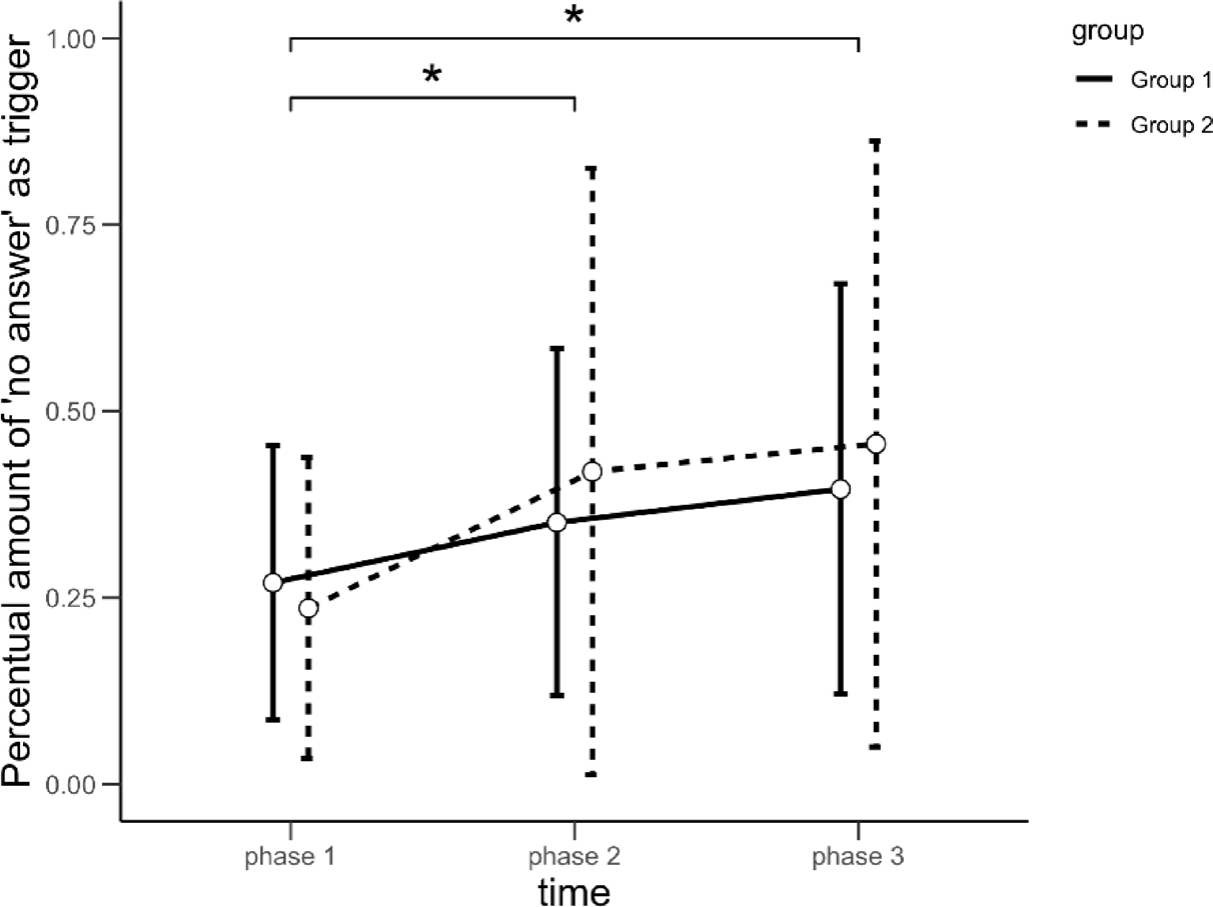
Changes in the percentage of trigger 1 (no answer) reported as stress-evoking event during the past five hours via EMA in the course of the study participation, differentiated by group. Group 1 received MBERT first before continuing with TAU, whereas group 2 received the MBERT after a TAU phase.

The performed rmMANOVA with the factors time and group revealed no significant within-subject time × group interaction (*Wilks λ* = 0.676, *F*(14,84) = 1.298, *p* > .05, *η*_*p*_*²* = 0.18) but a significant main effect of time (*Wilks λ* = 0.478, *F*(14,84) = 2.683, *p* < .01, *η*_*p*_^2^ = 0.31). This was composed of a significant main effect for trigger 1 (no answer) (Huynh-Feldt: *F*(1.519,36.462) = 9.936, *p*_*corr*_ < 0.01, *η*_*p*_*²* = 0.29), indicating that over time patients reported significantly more often that there had been no stress-triggering events in the past five hours. This main effect was characterized by a linear relationship (*F*(1, 24) = 13.157, *p* < .001, *η*_*p*_^2^ = 0.354). Post-hoc *t*-tests revealed significant increases in the report of no triggers (i.e., trigger 1) from phase 1 to phase 2 and from phase 1 to phase 3 (see Fig. 3). For detailed results of all post-hoc tests see Table S8 in the supplementary material. Note that the repeated-measures MANOVA was also calculated excluding triggers 7 and 8, which were rarely reported. The pattern of results remained the same, suggesting that the overall effects were not driven by these two triggers.

Finally, we investigated if the triggers differed in their impact on subjectively perceived stress and rumination. To this end, we performed another rmMANOVA for the factors time, group and trigger with the mean stress and rumination scores for each patient and each trigger during each phase. Note that if a patient didn’t report a specific trigger during a phase at all, the mean stress and rumination scores for this patient during this phase and for this trigger were treated as zero. Results revealed a highly significant constant term, indicating significant differences in the results for stress and rumination (*Wilks λ* = 0.087, *F*(2,23) = 120.985, *p* < .001, *η*_*p*_*²* = 0.91). Further, the rmMANOVA revealed a significant time × trigger interaction (*Wilks λ* = 0.864, *F*(28,670) = 1.821, *p* < .01, *η*_*p*_*²* = 0.07) as well as a significant time × group interaction (*Wilks λ* = 0.816, *F*(4,94) = 2.515, *p* < .05, *η*_*p*_*²* = 0.10). Additionally, the main effects for time (*Wilks λ* = 0.368, *F*(4,94) = 15.250, *p* < .001, *η*_*p*_*²* = 0.39) and for trigger (*Wilks λ* = 0.363, *F*(14,334) = 15.767, *p* < .001, *η*_*p*_*²* = 0.40) reached significance.

The time × trigger interaction reached significance in the case of stress (Huynh-Feldt: *F*(11.464,275.139) = 2.395, *p*_*corr*_ < 0.05, *η*_*p*_*²* = 0.09) but not rumination (Huynh-Feldt: *F*(11.302,271.241) = 1.531, *p*_*corr*_ > 0.05, *η*_*p*_*²* = 0.06). With regard to the interaction effect of time and group we found this interaction to yield significance for rumination (Huynh-Feldt: *F*(1.763,42.318) = 4.540, *p*_*corr*_ < 0.05, *η*_*p*_*²* = 0.159) but not for stress (*F*(2,48) = 0.596, *p*_*corr*_ > 0.05, *η*_*p*_*²* = 0.02). This interaction effect for rumination was characterized by a quadratic relationship (*F*(1,24) = 12.834, *p* < .01, *η*_*p*_*²* = 0.35). The main effects of time and trigger reached significance for both stress (main effect time: *F*(2,48) = 21.547, *p*_*corr*_ < 0.01, *η*_*p*_*²* = 0.47; main effect trigger: Huynh-Feldt: *F*(5.986,143.658) = 19.672, *p*_*corr*_ < 0.05, *η*_*p*_*²* = 0.45) and rumination (main effect time: Huynh-Feldt: *F*(1.763,42.318) = 40.806, *p*_*corr*_ < 0.01, *η*_*p*_*²* = 0.63; main effect trigger: Huynh-Feldt: *F*(5.899,141.574) = 18.862, *p*_*corr*_ < 0.05, *η*_*p*_*²* = 0.44). All of these main effects were characterized by a linear relationship (main effect time – stress: *F*(1,24) = 30.651, *p* < .001, *η*_*p*_*²* = 0.56; main effect time – rumination: *F*(1,24) = 54.244, *p* < .001, *η*_*p*_*²* = 0.693; main effect trigger – stress: *F*(1,24) = 13.431, *p* < .01, *η*_*p*_*²* = 0.36; main effect trigger – rumination: *F*(1,24) = 35.282, *p* < .001, *η*_*p*_*²* = 0.59) as well as, with exception for the main effect of time in the case of stress, a quadratic relationship (main effect time – rumination: *F*(1,24) = 5.070, *p* < .05, *η*_*p*_*²* = 0.17; main effect trigger – stress: *F*(1,24) = 59.331, *p* < .001, *η*_*p*_*²* = 0.71; main effect trigger – rumination: *F*(1,24) = 27.713, *p* < .001, *η*_*p*_*²* = 0.54). The main effect of trigger in the case of rumination additionally was characterized by a cubic relationship (*F*(1,24) = 5.640, *p* < .05, *η*_*p*_*²* = 0.19).

Taken together, these results indicate that patients reported fewer triggers overall over the course of the study, independent of group assignment (see above). Moreover, even when stress-inducing triggers were reported, their impact on subjective stress and rumination decreased over time: while different triggers generally had less impact on subjective stress with ongoing study participation, they elicited significantly less rumination during the MBERT compared to the TAU phase of the study. Detailed results of all statistical analyses including post-hoc comparisons, which were kept concise here for readability, can be found in the supplementary material (see Table S8, Table S9, Table S10, Table S11 and Figure S1).

## Discussion

The study at hand investigated the effectiveness of an MBERT in the treatment of MDD, using data from patients’ everyday lives, assessed via EMA. This was achieved by examining general changes in therapy-related variables, namely subjective stress, rumination, self-efficacy, self-kindness, mindful distancing, and sleep quality, as well as by analyzing the temporal dynamics of those changes. Additionally, different triggers were examined concerning their impact and changes in the amount of this impact on stress and rumination.

In line with the already found efficacy of MBERT in reducing depressive symptoms and rumination as well as increases in self-efficacy and self-compassion^39^, we found that depressed patients reported less stress and rumination and more self-efficacy, self-kindness and mindful distancing as well as an improvement in sleep quality at the end of the study compared to the beginning. Even though these results may not be surprising at first glance, it is particularly noteworthy that by using EMA, we were able to demonstrate that the described effects are not limited to the therapeutic context, but also manifest in patients’ daily lives. This is crucial, as patients might feel more secure in contact with their therapist and in the rooms where they received the therapy (context effect), possibly leading to reports of stronger improvements than they might be outside of this context. Furthermore, EMA likely reduced the probability of socially desirable responses. Taken together, the described results strengthen the assumed effectiveness of MBERT and show that the treatment effects even have an impact on well-being in patients’ daily lives. Furthermore, these effects remained stable over a period of approximately four weeks after completion of MBERT, as demonstrated by the wait-list control design. Examined in more detail, we found continuously perceived reductions in stress and rumination as well as increases in self-efficacy and mindful distancing over the course of the study participation. Again, as in the analyses of pre-post comparisons and in situ effects^39^, the reduction of rumination as well as the increases in self-efficacy and mindful distancing and even in self-kindness and sleep quality were stronger when patients received MBERT compared to TAU. Interestingly, this was the case even though subjectively experienced stress decreased continuously overall, but there was no difference between MBERT and TAU. Consequently, the previously described changes took place when receiving psychotherapy despite a comparable level of stress to that without a psychotherapeutic treatment, leading to the conclusion that patients really seem to learn strategies and gain resilience through psychotherapy, facilitating their daily lives even when confronted with (similar amounts of) stress. These improvements in daily life were likely driven by the skills learned through MBERT, such as enhanced mindfulness, reappraisal, and distancing from distressing emotions and thoughts. These skills appear to have contributed directly to the reductions in stress and rumination, as well as the increases in self-efficacy, mindful distancing, self-kindness, and sleep quality observed during the study. These mechanisms align with theoretical models of rumination, particularly the S-REF model^33^ and the attentional scope model. MBERT may help interrupt maladaptive self-focused processing (S-REF) and widen attentional scope^34^ through mindful awareness and acceptance. The structured four-step process also addresses abstract, evaluative thought patterns^77^ by guiding patients toward more concrete and emotionally grounded engagement with distressing content. Thus, MBERT seems to target core cognitive processes implicated in the maintenance of rumination.

With regard to the specific triggers that elicited stress and rumination in patients with MDD, we found that private obligations and internal causes were experienced as the most stress-inducing before/at the beginning of the study. Rumination was most strongly triggered by internal causes, which is in line with different theories claiming negative mood and negative emotions as well as negative thoughts about oneself (e.g., about one’s own symptoms, weaknesses and failures) as possible triggers for rumination (e.g.,^78,79^). Throughout the study participation these effects diminished, i.e., patients reported significantly more often that there hadn’t been any stressful triggers at all (i.e., no triggers were reported) and if they did report a trigger, these led to less stress and rumination. However, internal causes still were the most stress-eliciting trigger even at the end of the study. These results are well in line with the previously described increases in self-efficacy, self-kindness and mindful distancing as well as the reduction of depressive symptoms in general: Patients reported fewer triggers while they most probably might have had comparable numbers of appointments etc., leading to the conclusion that similar situations were experienced as less stressful due to the treatment. Furthermore, if reported, the triggers led to lower subjective stress and rumination, demonstrating an improved ability to cope with such everyday challenges. However, when interpreting this post-treatment data, it also needs to be considered that triggers in general were reported much less often at the end of the study, demonstrating an alignment of the responses of the MDD sample to those of the healthy samples in our previous studies^26,51^ where significantly fewer triggers were reported by healthy controls (HC) than by MDD patients. This alignment towards the HC sample again supports the effectiveness of the treatment. Overall, the findings confirm that social interactions (and internal causes) play a major role as triggers of stress and rumination in patients with MDD^26,51^. These results further underline the importance of topics like stress management and training of social competencies in the treatment of MDD^80^, as it is already well established in many (especially cognitive behavioral) psychotherapeutic treatments (e.g.,^81–84)^.

In the exploratory analysis examining the temporal dynamics of the previously described changes, we found that ruminative behavior predicted lower levels of subsequent self-efficacy and self-kindness. However, since rumination decreased and both self-efficacy and self-kindness increased over time (see above), this may indicate increased robustness and/or more effective strategies to interrupt ruminative processes, such that their negative impact on self-efficacy and self-kindness no longer inhibited therapeutic progress overall. While lower levels of mindful distancing were marginally associated with higher rumination later on, higher levels of mindful distancing predicted subsequent increases in both self-efficacy and self-kindness. Furthermore, self-kindness, self-efficacy, and mindful distancing appeared to reinforce each other over time and were associated with lower levels of subjectively perceived stress. These findings are in line with previous literature highlighting mindfulness as a key mechanism of change in (mindfulness-based) psychotherapy^44–48^ and underscore its relevance in contemporary therapeutic approaches such as third-wave CBT (e.g., mindfulness-based cognitive therapy, MBCT^85^; Acceptance and Commitment Therapy, ACT^38^). The reciprocal relationships between self-kindness, self-efficacy, and mindful distancing suggest that these variables change more or less commonly, which leads us to the conclusion that such cognitive therapeutic improvements do not follow a clear, sequential pattern like individual dominoes knocking each other over, but rather co-develop in a mutually reinforcing dynamic – similar to different sides of a die, all moving together when one side is pushed. However, these improvements not only appear to be facilitated by effective psychotherapeutic interventions, as Bandura already emphasized^42,43^, but furthermore themselves seem to play an influencing role in the change of other related variables, namely subjective stress experiences. Notably, while several main effects were observed across study phases, some associations, namely between higher self-efficacy, self-kindness, mindful distancing and lower stress, as well as between self-efficacy and self-kindness, were significantly stronger during MBERT. This suggests that MBERT may have enhanced these dynamic processes and their contribution to stress regulation. However, other observed effects, namely higher self-efficacy and mindful distancing following higher self-kindness as well as higher mindful distancing itself leading to higher self-efficacy and self-kindness later on, occurred independently of the study phase, indicating that these changes were not exclusive to MBERT and may also reflect general therapeutic or time-related processes, including those occurring during TAU. Taken together, the findings point to the importance of these transdiagnostic psychological processes in both standard care and structured psychotherapy. While MBERT may have specifically amplified some of these effects, especially in reducing subjective stress through improved self-efficacy, self-kindness, and mindful distancing, the overall pattern suggests a broader dynamic in which these variables evolve together and influence coping processes over time.

A few limitations should be considered. First of all, it should be noted that the analyses conducted have an exploratory character, which explains why the hypotheses examined in this paper do not appear in the preregistration of the study on ClinicalTrials.gov. Furthermore, the interpretation of the results is limited not only by additional challenges (see below) but also by the overall small sample size. With regard to the performed time-lagged segment analyses it must be considered that they may not fully capture the actual temporal dynamics of change. In the analyses we only considered lags of one assessment point, whereas one could also investigate predictions over longer periods of time, e.g., a week. However, the statistical models quickly become very complex and thus difficult to interpret^86^ and the model with a lag of 1 was often found to have the best fit to the data^69,70,87^. Possibly, this might be due to greater similarities between data close together in time compared to data further apart in time. However, as with the study design we explicitly wanted to investigate the effects under ecologically valid circumstances, it is also to be expected that possible influential effects of the assessed variables are only of short duration. Also, the dynamics found surely are not influenced by MBERT solely but also by everyday events. In this context, it must also be taken into account that more than half of the patients received additional outpatient psychotherapy and/or medication treatment during their study participation, with some experiencing changes such as starting or discontinuing a treatment. This may have influenced their individual emotion regulation and ruminative behavior. However, the randomized groups did not differ in terms of the amount or type of such additional psychotherapeutic or pharmacological treatments, and therefore, these are unlikely to have had a significant impact on the results. The investigation of the mechanisms of action and segment effects of psychotherapy is further complicated by the fact that there are many different factors that also influence therapeutic processes and effects, but are difficult to measure and control in studies, such as the therapeutic alliance (e.g.,^ 68,88^). Finally, the question of the actual mechanisms of action that contributed to improvement must be answered individually for each patient, as the causes and reasons for being depressed are unique and thus every psychotherapeutic treatment needs to be adapted individually. One possible way to account for such individual factors could be to assess how consistently therapeutic strategies and homework assignments are implemented between sessions and to incorporate this as a covariate. Likewise, in future studies investigating the effects of psychotherapeutic treatments in patients’ daily lives, both types of variables could be respected: those consciously related to psychotherapy (such as thoughts about the last session and the use of strategies) and those that rather capture symptoms and general mental well-being, as we have assessed.

Despite these limitations, the results make an important contribution to the question of the mechanisms of action and temporal dynamics of psychotherapy in depression. We were able to demonstrate, in particular, that the treatment also affects patients’ daily lives in a desired way, and we substantiated this through a large amount of data in a relatively large sample (compared to other treatment studies), as well as the waiting-list randomized controlled design. We also demonstrated that EMA is an effective tool for assessing such (therapeutic) effects in ecologically valid settings.

In conclusion, we demonstrated that the effectiveness of MBERT could not only be seen in pre-post comparisons but also continuously in patients’ daily lives. In doing so, the temporal changes in self-efficacy, self-kindness, and mindful distancing appear to go hand in hand, reducing the perception of stress later on, which provides valuable evidence for further considering these variables as a motor of change in psychotherapy and paving the way to an enhanced psychotherapy. Furthermore, this is additionally supported by increased resilience against stress-eliciting triggers throughout psychotherapy, leading to less ruminative behavior and stress even when stressful life events may occur.

## Materials and methods

### Participants

Participants were recruited via emails and flyers at the University Hospital of Tuebingen, the University of Tuebingen and through outpatient psychotherapists. All procedures were approved by the ethics committee of the University and University Hospital of Tuebingen and were in line with the Declaration of Helsinki in its latest version. The study protocol is registered at ClinicalTrials.gov (NCT04560192; date of first registration: 23/09/2020). Prior to data collection, all participants gave written informed consent. Exclusion criteria for participation in the study included age younger than 18 or older than 60 years, prior participation in studies with a Trier Social Stress Test (TSST) or a similar procedure and, on a somatic level, kidney insufficiency, diabetes mellitus, cushing syndrome, adrenal insufficiency, dysrhythmia, hypertension, pacemaker, cortisone medication, and craniocerebral trauma. Further, any primary mental or personality disorder, including substance abuse (current as well as remitted), led to exclusion, except for a current primary F32.x, F33.x and F34.1 ICD-10 diagnosis in the MDD sample. Regarding this MDD sample, patients with extraordinarily severe depressive symptoms (Beck Depression Inventory II (BDI-II)^89^: score > 50), acute suicidality, and strong decompensation under social stress in the past were not included in the study due to the stressful TSST procedure. The group of HC was composed of participants with low trait rumination (Ruminative Response Scale (RRS)^90^: score ≤ 2).

**Fig. 4:**
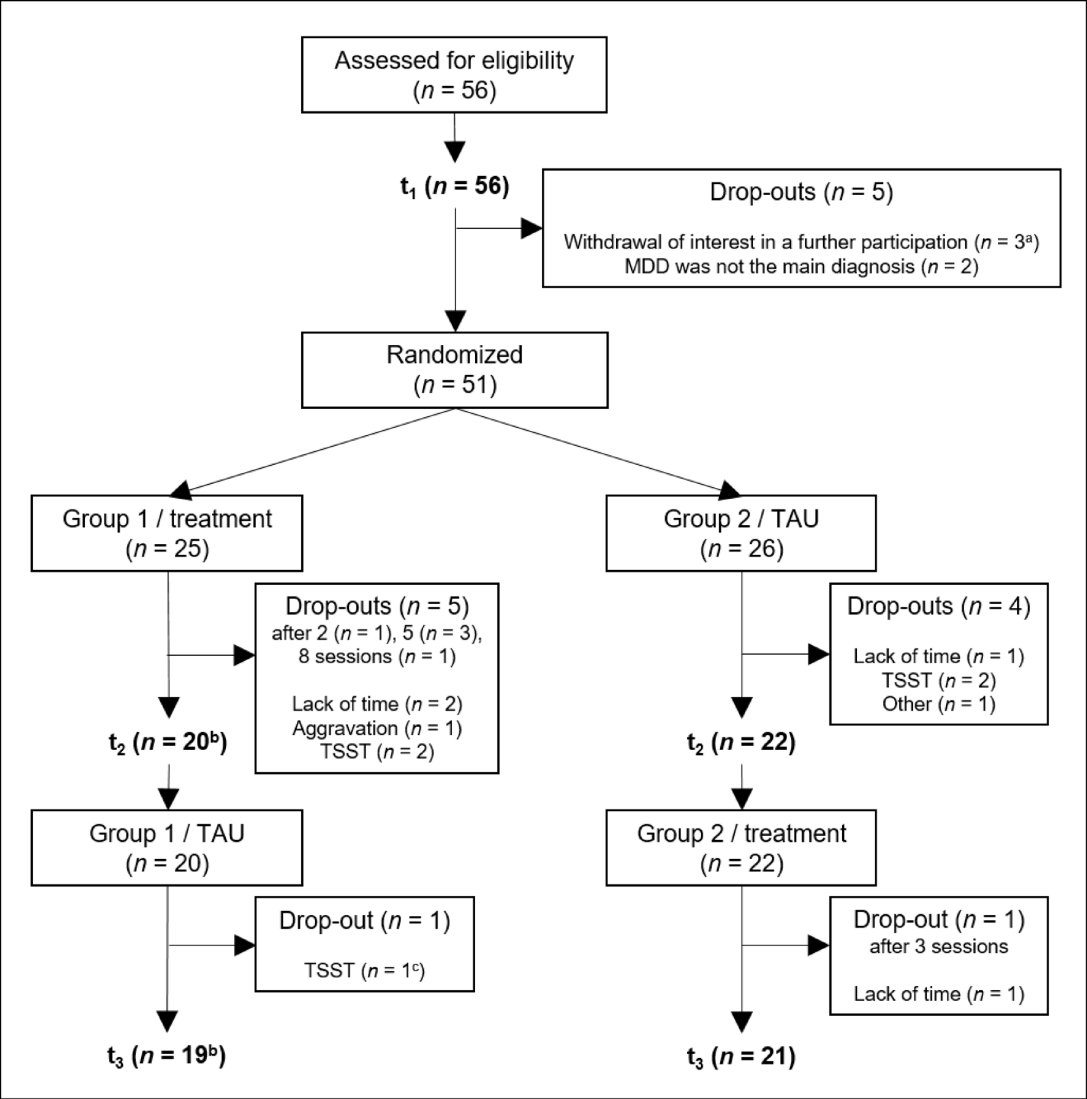
Flow of participants. *Note.* TSST = unwillingness to participate in another stress paradigm later in the study. ^a^One patient asked all collected data to be deleted. ^b^The EMA data of one patient could not be used in the analyses due to technical issues (data entries incomplete; too few data entries in total). ^c^One patient did not participate in t_3_ but answered enough EMA and fulfilled all of the eight therapeutic training sessions, so its data could be used in the analyses.

We collected data of 56 patients with MDD and of 43 HC. As only patients in the experimental group were asked to answer EMA, only the data of this clinical sample is analyzed in the present article. Due to dropouts and difficulties in EMA data collection, data of only 40 patients could be included in the analyses (see Fig. 4). This analyzed sample did not differ in age, sex, symptom severity, diagnosis, comorbidities, self-efficacy, self-compassion, or state rumination from the dropouts (for a more detailed description of the sample characteristics of the dropouts/excluded patients see supplementary material Table S2). Within the analyzed sample, primary diagnoses included recurrent (*n* = 38) as well as first episode (*n* = 2) MDD, and comorbid diagnoses included anxiety disorder (e.g., social anxiety disorders, specific phobias such as acrophobia) (*n* = 15), remitted eating disorders (*n* = 4) and personality disorders (*n* = 2).

The average BDI-II score at the beginning of the study was 26.34 (*SD* = 7.86). The mean age of the analyzed sample was 32.73 years (*SD* = 11.08 years) and 67.5 % of the patients were female. Twenty-one of the analyzed patients (52.5 %) had already received psychotherapeutic treatment(s) in the past. At the beginning of their study participation 45 % were currently receiving psychotherapy and another 42.5 % were currently using antidepressant medication. However, four patients stopped their outclinic psychotherapy during their study participation, whereas another four patients started one. Further, two patients began with an antidepressant medication, whereas another four patients stopped their initial antidepressant medication. The groups did not differ in terms of the amount or type of psychotherapeutic or pharmacological treatment received alongside the study (see also supplementary material Table S3).

### Procedures

All participants participated in a first TSST. Afterwards, MDD patients were block-randomized (counterbalanced for sex and severity of depressive symptoms using Microsoft Excel, depending on the corresponding values determined at the first TSST) into either MBERT (Group 1; *n* = 19) or treatment as usual (TAU) (Group 2; *n* = 21). The MBERT consisted of a psycho-educational session aimed at providing an understanding of the relationship between negative emotions and rumination, followed by eight individual training sessions using a four-step ERS, based on current issues patients were ruminating about. This ERS was structured as follows: After identifying the primary emotion in the topic or conflict, the steps were (1) conscious concentration, (2) benevolent acceptance, (3) cognitive reevaluation/reinterpretation, and (4) distancing from the emotion. Each training session was conducted individually and lasted approximately 60 to 90 min. To enable parallel acquisition of in situ neural data, the MBERT was adapted for concurrent functional near-infrared spectroscopy (fNIRS) recordings, i.e., cortical oxygenation was continuously assessed during each session. For this purpose, each session was divided into 40 block-randomized trials of 40 s each, consisting of 20 active training trials and 20 pause trials. During active trials, patients were asked to apply the ERS (completing several trials for each of the four steps); during pause trials, they were instructed to not focus on the current topic but to let their thoughts wander freely. This structure allowed for a reliable differentiation of neural activation patterns between regulation and rest conditions in the analysis of the fNIRS data. Although the training sessions were not conventional psychotherapy sessions in the traditional sense, brief therapeutic conversations were held between trials as needed to provide individual guidance and support the implementation of the ERS (for more details see^39^). Note that the TAU group was treated as an active wait-list control group as ongoing psychotherapeutic and pharmacological treatments were allowed to be continued and furthermore all patients were allowed to initiate (or stop) such treatments on their own accord during study participation. After this first block of MBERT or TAU, which lasted approximately four to five weeks, all patients participated in another TSST. Afterwards, following a block-randomized controlled design, groups switched, so the initial treatment group now was the active control group (TAU) and vice versa, before all patients participated in a final TSST. A whole study participation lasted approximately ten weeks. As during all training sessions, cortical oxygenation was also assessed during all TSSTs via fNIRS. Note that these and further (in situ) results of the MBERT as well as the results of the TSST measurements are reported elsewhere^39^ and that all data analyzed in this article were collected independently of the fNIRS measurements. For more detailed descriptions of the procedures of the TSST and MBERT, please see^39^. At the time of randomization, groups did not differ in symptom severity, self-efficacy, or rumination (BDI-II: *t*(38) = 0.643, *p* > .05, *d* = 0.204; Self-efficacy (Skala zur Allgemeinen Selbstwirksamkeitserwartung, SWE)^91^: *t*(38) = -0.996, *p* > .05, *d* = 0.316; rumination (state rumination questionnaire, SRQ): *t*(38) = -0.475, *p* > .05, *d* = 0.150) but they did differ in self-compassion (Self-Compassion Scale (SCS-D)^92^: *t*(38) = 2.699, *p* < .05, *d* = 0.855), with a higher initial self-compassion in group 2 (*M* = 24.786) than in group 1 (*M* = 23.263).

With completion of the first and until completion of the third TSST, all MDD patients, regardless of their randomization, were asked to answer a questionnaire via EMA twice per day (midday and evening). This was realized by using the PsyAssesor researcher edition V2 (Machine Learning Solutions, Luxembourg, 2019). After the first log-in, patients were asked to define two times per day, at which they wanted to answer the EMA. The first of those was supposed to be approximately at noon but at least five hours after getting up in the morning. The second assessment time each day was supposed to be approximately six hours later. Theoretically, the set times could be changed at any point during the course (e.g., if a patient’s daily routine changed significantly). Patients got a reminder email five minutes before every assessment and were asked to additionally install an alarm clock on their phones for the set times of the assessment. At each assessment, patients had a time window of 30 min to answer the EMA. In detail, patients were asked to rate their subjective stress level regarding the past five hours using a slider (0 % = not at all; 100 % = very much) and to report possible stressful life events during these five hours as a free text. Furthermore, they were asked to rate their current amount of ruminative processes using three modified items of the RRS and three modified items of the Perseverative Cognitions Questionnaire (PCQ^93^), respectively. The RRS was selected for consistency with the measures of rumination during the TSST measurements despite the existence of validated EMA measures of rumination (e.g.,^94^). Additionally, momentary perceived self-kindness was assessed using three modified items of the SCS-D and the current levels of self-efficacy and mindful distancing were assessed via three adapted items (e.g., from the Generalized self-efficacy scale (GSE)^95^), respectively. Answers were given using a slider that ranged from 1 (not at all) to 5 (very much) with exception for self-efficacy, where the slider ranged from 0 (not at all) to 100 (very much). The current mood was rated using a circumplex on the axes arousal (aroused vs. relaxed) and valence (positive vs. negative). Finally, patients reported how recovered they felt after their last sleep on a scale from 1 (not at all) to 5 (totally recovered) using a slider. A more detailed description of the used items as well as the original questionnaires they were taken from is to be found in the supplementary material (Table S1). The time frame of the last five hours was chosen to interpret the responses (e.g., extent of rumination or self-efficacy) in a plausible context with potentially occurring stress-inducing events and to avoid carry-over effects (e.g., from the previous evening). Data were saved under an individual pseudonym for each patient. Via their account on the website, patients could always see what percentage of all possible assessment points they had completed. They were encouraged to fill out at least 80 % of the assessment points and were reminded by the study therapist (first author) if they did not fill out the EMA regularly enough. One patient did not own a smartphone, so the assessment forms were printed out and given to this patient, allowing him/her to fill them out manually. Afterwards, they were added to the data table manually.

### Data preparation and analyses

Since we used self-created items in the assessment of self-kindness and mindful distancing, and these were highly interrelated due to repeated measures, we conducted an exploratory multilevel factor analysis in MPlus across all items of the proposed scales on rumination, self-efficacy, self-kindness and mindful distancing to check whether the assumption of four different scales is justified. Next, we checked for sufficient data variability in each patient and each dependent variable, calculating the variances and excluding patients with variances of zero. However, no patient needed to be excluded from further analyses. Additionally, we calculated repeated measures correlations^75,76^ between all dependent variables. To investigate changes in the variables from the beginning to the end of the study participation, with additional respect to the time point when groups switched (“halftime”), we calculated baseline, halftime and endpoint values for each variable. For the baseline values, we took the first seven data entries of each patient and calculated the means and the standard deviations for each dependent variable out of this reduced dataset. We did the same with the first seven data entries after completion of the last training session in group 1 (receiving MBERT before TAU) as well as with the last seven data entries before receiving the first training session in group 2 (receiving MBERT after TAU) to calculate mean values for the time point when groups switched (“halftime”). Finally, we did the same with the last seven data entries of each patient to calculate endpoint values. The number of seven data entries was chosen as there was a minimum delay of four days between the first TSST and the first training session. Data collection via EMA started immediately after the first TSST for all patients twice per day so that no patient should have received any therapeutic intervention during the first seven assessment points. For reasons of better comparison, we equaled the number of data entries for the halftime and endpoint statistics to those of the baseline statistics. We then conducted a repeated measurement multivariate analysis of variance (rmMANOVA) for the factors group (Group 1 vs. Group 2) and time (baseline, halftime, endpoint). The Huynh-Feldt correction was used in cases of violated sphericity^96^. For the analysis of post-hoc comparisons, pairwise (within groups) and independent (between groups) *t*-tests were performed. To correct for multiple testing, the Benjamini-Hochberg procedure was used^97,98^. A detailed presentation of the results including corrected as well as uncorrected *p*-values is given in the supplementary material.

Next, linear mixed models were used to investigate the impact of study phase (MBERT vs. TAU) and time on each dependent variable and to perform time-lagged segment analyses in the analysis of temporal dynamics of change, allowing for an investigation of the influence of a predictor variable at time t, controlled for t − 1, to an outcome variable at t + 1, controlled for t. Like that, time-lagged segment analyses ensure that there are no overlaps in time between the specific segments, allowing for interpretations of the direction of effects and of temporal associations between the variables^67^. In contrast to the rmMANOVA investigating pre-post comparisons (see above), all individual data points were used in these linear mixed models to also take into account individual fluctuations.

The causes of the subjective stress levels patients reported via free text were categorized by two independent raters, using the following categories: (1) no answer, (2) social interaction, (3) work, (4) private obligations, (5) daily hassles, (6) internal causes, (7) sleep quality, and (8) political events (for information on the definition of these causes, see supplementary material). The inter-rater reliability was very high (Cohen’s *κ* = 0.841, z = 111.91, *p* < .001). For the analysis of differences in the occurrence of those triggers, we defined the data within the first 14 days after the first TSST as phase 1, the data within the last seven days before and the first seven days after the second TSST as phase 2 and the data within the last 14 days before the third TSST as phase 3. Patients who missed more than 50 % in one or more of these phases of EMA were excluded from further analyses. Then the percentage of each trigger within each patient and each phase was calculated. This allowed us to perform a rmMANOVA for the factors group (Group 1 vs. Group 2) and time (phase 1 vs. phase 2 vs. phase 3). Furthermore, mean scores of subjective stress and rumination were calculated for each trigger and each patient within each phase and another rmMANOVA was performed for both variables, with the factors group, time and trigger. Again, the Huynh-Feldt correction was used to correct for violated sphericity^96^ and the Benjamini-Hochberg correction to correct for multiple testing^97,98^. Note that polynomial contrasts of higher than cubic order will not be reported as they most likely resulted from spurious fluctuations.

Data analyses were done using R^99^, SPSS^100^ and MPlus^101^. We calculated mixed models using the R-packages lme4^102^ and lmerTEST^103^. For calculating marginal *R*^*2*^ as a measure of variance explained by the fixed effects in the mixed models, the R-package MuMIn^104^ was used.

## Supplementary Information

Below is the link to the electronic supplementary material.

Supplementary Material 1

## Data Availability

The datasets used and/or analyzed during the current study are available from the corresponding author (Hendrik Laicher; mail: hendrik.laicher@med.uni-tuebingen.de) or the last author (David Rosenbaum; mail: david.rosenbaum@med.uni-tuebingen.de) on request.
