# Supplementary material for: Temporal dynamics of CBT emotion regulation training in patients with major depression using ecological momentary assessment
Source: Sci Rep. 2025 Aug 30;15:31992. doi: 10.1038/s41598-025-16344-3 (PMC12398587; doi:10.1038/s41598-025-16344-3)
Supplement: Supplementary file 1 — Supplementary Material 1 [file 41598_2025_16344_MOESM1_ESM.docx]

**Supplementary material:**

**Temporal dynamics of CBT emotion regulation training in patients with major depression using ecological momentary assessment**

Hendrik Laicher^1,2^, Isabell Int-Veen^1,2^, Leonie Woloszyn^1,2^, Ariane Wiegand^1,2^, Agnes Kroczek^1,2^, Daniel Sippel^1,2^, Glenn Lawyer^4^, Florian Torka^1,2^, Vanessa Nieratschker^1,2^, Julian Rubel^5^, Andreas Fallgatter^1,2,6^, Ann-Christine Ehlis^1,2,6^, David Rosenbaum^1,2^

^1^ Department of Psychiatry and Psychotherapy, University Hospital of Tuebingen, Tuebingen, Germany

^2^ Tuebingen Center for Mental Health

^3^ Department of Psychiatry, University of Muenster, Muenster, Germany

^4^ Machine Learning Solutions, Luxembourg, Luxembourg

^5^ Psychotherapy Research Lab, Psychology and Sport Sciences, Justus-Liebig-University Giessen, Giessen, Germany

^6^ LEAD Graduate School & Research Network, University of Tuebingen, Tuebingen, Germany

**Corresponding Author:**

Hendrik Laicher, Calwerstraße 14, 72076 Tuebingen, Germany

email: hendrik.laicher@med.uni-tuebingen.de

Phone: 00497071 29-87103

**Keywords:** Ecological Momentary Assessment (EMA), Major Depressive Disorder (MDD), Rumination, Repetitive Negative Thinking (RNT), Emotion Regulation (ER), time-lagged segment analyses

**Supplementary Introduction**

**Rumination – A maladaptive Emotion Regulation Strategy?**

Emotions themselves are difficult to define, and there is no universally accepted definition. However, most definitions agree that emotions include cognitive components (e.g., appraisals) as well as physiological and behavioral responses [1]. Emotion regulation, in turn, is typically defined as the processes aimed at influencing the onset, intensity, or duration of an emotional response (e.g. [2]). This consequence-based definition, however, raises conceptual issues. To illustrate this, consider the analogy of climate regulation: If we define “regulation” solely by its effects, we would have to classify cloud formation – which influences the climate but occurs naturally and without intention – as a form of regulation. In contrast, intentional human actions, such as reducing CO₂ emissions, are clearly regulatory. Most would agree that only the latter constitutes actual climate regulation, as it is goal-directed and intentional. The same distinction should apply to emotion regulation: Not every process that alters emotions is necessarily regulatory in nature. This distinction suggests the need to differentiate between ‘I regulate’ (intentional control) and ‘something is regulated’ (as a byproduct of other processes). According to a purely consequence-oriented definition, even involuntary physiological responses or automatic cognitions could be considered emotion regulation, simply because they affect emotional states (e.g., crying can alleviate sadness, even if it happens involuntarily). But this blurs the conceptual boundary between emotional experience and regulation. Rumination shares many features with such automatic responses: It often unfolds involuntarily, is experienced as uncontrollable and unwanted, and is described by patients as something that happens to them rather than something they actively do (e.g. [3, 4]). In the Dialectical Behavior Therapy (DBT [5]), ERS are defined more strictly: They must be aimed at reducing emotional burden by acting against the emotional impulse. Rumination, in contrast, follows the emotional impulse and is affect-congruent – individuals repeatedly think about the emotional content and its triggers rather than working to reduce the distress. In this light, rumination appears not as a failed or maladaptive attempt at (down-) regulation, but as a continuation of the emotional episode itself. We therefore argue that rumination should not automatically be classified as an ERS. Instead, it may be more acurately conzeptualized as either (a) a cognitive component or manifestation of an (unresolved) emotional state [1], or (b) a consequence of failed or absent emotion regulation. In fact, some items in rumination questionnaires might simply capture such secondary appraisals of emotion, e.g., “Can I cope?”, “Why do such things always happen to me?” or “Why do I always feel this way?”. These thoughts point to an ongoing emotional struggle rather than an effort to resolve it. Accordingly, we suggest refining the definition of emotion regulation by placing greater emphasis on intention, goal-directedness, and (in case of down-regulation) emotion-incongruence of the reaction. Rumination lacks these features. This theoretical ambiguity highlights the importance of continued theoretical and empirical work to clarify whether rumination is best understood as a regulatory strategy, a cognitive component of emotion, or a marker of dysregulated affect.

**Supplementary Methods**

**Items used in the EMA to assess rumination, self-efficacy, self-kindness and mindful distancing**

Table S1
*Items used in the assessment of self-efficacy, rumination, self-kindness and mindful distancing twice per day via ecological momentary assessment (EMA). The items are listed in the order in which they were presented in the EMA.
^a^If no source is listed, the item was self-created, but adapted from existing questionnaires (e.g. General Self-Efficacy Scale (GSE) [6]). For validation via factor analysis see main article. PCQ = Perseverative Cognitions Questionnaire [7], RRS = Ruminative Response Scale [8], SCS-D = German version of the Self-Compassion Scale [9].*

| **no.** | **German translation** | **English translation** | **Source^a^** |
| --- | --- | --- | --- |
| *Self-efficacy* | | | |
| 1 | Ich war davon überzeugt, dass ich die Mittel und Fähigkeiten besitze, etwas an meiner Situation zu verändern. | I was convinced that I possess the means and abilities to change something about my situation. |  |
| 2 | Ich hatte den Eindruck, dass ich meine emotionalen Zustände selbst beeinflussen kann. | I had the impression that I could influence my emotional states myself. |  |
| 3 | Ich hatte den Eindruck, dass ich durch das, was ich tue, meine Situation verändern kann. | I had the impression that I could change my situation through what I do. |  |
| *Rumination* | | | |
| 1 | Ich dachte wiederholt über Dinge nach, die längst vorbei waren. | I repeatedly think about things that are over and done with. | PCQ, Item 23 |
| 2 | Ich fand es schwierig, einen Gedanken loszulassen, wenn er einmal in meinem Kopf war. | Situations that have happened often drift back into my mind. | PCQ, Item 2 |
| 3 | Ich spielte wiederholt vergangene Geschehnisse in meinem Kopf durch. | I repeatedly play back past events in my mind. | PCQ, Item 20 |
| 4 | Ich dachte an all meine Misserfolge, Defizite und Macken. | Think about all your shortcomings, failings, faults, mistakes. | RRS, Item 18 |
| 5 | Ich fragte mich, warum ich Probleme habe, die andere nicht haben. | Think “Why do I have problems other people don’t have?” | RRS, Item 15 |
| 6 | Ich dachte darüber nach, warum ich die Dinge nicht besser in den Griff bekomme. | Think “Why can’t I handle things better?” | RRS, Item 16 |
| *Self-kindness* | | | |
| 1 | Ich akzeptierte meine Fehler und Schwächen. | I was tolerant of my own flaws and inadequacies. | SCS-D, Item 23 |
| 2 | Ich schenkte mir selbst die Zuwendung und Einfühlsamkeit, die ich brauchte. | I gave myself the caring and tenderness I needed. | SCS-D, Item 12 |
| 3 | Ich versuchte, verständnisvoll und geduldig mit mir selbst zu sein. | I tried to be understanding and patient with myself. | SCS-D, Item 26 |
| *Mindful distancing* | | | |
| 1 | Ich konnte mich von meinen Gedanken und Gefühlen abgrenzen. | I was able to separate myself from my thoughts and feelings. |  |
| 2 | Ich konnte meine Gedanken und Gefühle nüchtern betrachten. | I was able to view my thoughts and feelings objectively. |  |
| 3 | Ich nahm meine Gefühle und Gedanken wahr, ohne diese als gut oder schlecht zu bewerten. | I observed my feelings and thoughts without judging them as good or bad. |  |

**Definitions of stress-evoking life events**

In the inter-rater evaluation of the events reported as stressful by patients in the EMA, the following categories were used:

1. No answer (no stressful event / nothing reported)
2. Social interaction (job-related / private; perceived positively / negatively; e.g. meeting somebody, having an argument with somebody)
3. Work (job, studies; e.g. deadlines, workload)
4. Private obligations (i.e. not job-related appointments and tasks; e.g. appointment with a doctor, therapy, hobbies)
5. Daily hassles (e.g. daily routines, going shopping, planning and organization, traveling, parenting)
6. Internal causes (personal thoughts and feelings; e.g. thoughts, worries, decisions, pain, misconducts, time pressure, illness)
7. Sleep quality (e.g. difficulties with falling asleep, insomnia, dreams, oversleeping)
8. Political events (e.g. coronavirus, world affairs)

**Comparison of the included and the excluded sample**

Table S2
*Sample characteristics (mean (standard deviation)) and analytic comparisons between the patient included in and excluded from the analyses.
BDI-II = Beck Depression Inventory II [10]; SWE = Skala zur Allgemeinen Selbstwirksamkeitserwartung [11]; SCS-D = Self-Compassion Scale [9]; SRQ = State Rumination Questionnaire.
^a^Reasons for exclusion included dropouts and difficulties in EMA data collection. ^b^As one patient asked all his / her data to be deleted, it could not be respected here, resulting in a sample size of n = 15. ^c^Due to missing data in one subject, the respected sample size in the excluded sample regarding the SCS-D data is n = 14.*

| Variable | Included sample *(n = 40)* | Excluded sample^a^ *(n = 15^b^)* | Analytical comparison |
| --- | --- | --- | --- |
| Age | 32.73 years (11.08 years) | 32.27 years (11.61 years) | *t*(53) = 0.135, *p* > .05, *d* = .041 |
| Sex | 67.5 % female | 73.3 % female | *t*(53) = 0.410, *p* > .05, *d* = .124 |
| Diagnosis | first episode MDD: *n* = 2 recurrent MDD: *n* = 38 | first episode MDD: *n* = 1 recurrent MDD: *n* = 14 | *χ*^2^(1) = 0.059, *p* > .05, *V* = .808 |
| Comorbidity | Anxiety disorder: *n* = 15 remitted eating disorder: *n* = 4 personality disorder: *n* = 2 | Anxiety disorder: *n* = 7 remitted eating disorder: *n* = 1 personality disorder: *n* = 2 remitted substance abuse: *n* = 1  ADHD: *n* = 1 | Wilks λ = 0.869, *F*(5,48) = 1.451, *p* > .05, *ηp²* = 0.131 |
| BDI-II | 26.34 (7.86) | 27.367 (5.658) | *t*(53) = -0.463, *p* > .05, *d* = .140 |
| SWE | 24.063 (4.825) | 24.133 (5.208) | *t*(53) = -0.047, *p* > .05, *d* = .014 |
| SCS-D | 2.163 (0.334) | 2.261 (0.625) | *t*(52c) = -0.742, *p* > .05, *d* = .230 |
| SRQ | 2.813 (0.795) | 2.693 (0.764) | *t*(53) = 0.503, *p* > .05, *d* = .152 |

**Comparison of the included and the excluded sample**

Table S3
*Sample characteristics and analytical comparisons between the groups regarding the amount of psychotherapeutic and antidepressant pharmacological treatment. CBT = Cognitive Behavioral Psychotherapy, PD = Psychodynamic Psychotherapy.
^a^One of those two patients was one that stopped his / her initial psychotherapeutic treatment between the first and the second TSST and then started a new one between the second and the third TSST.*

| time | Group 1 (MBERT 🡪 TAU) | Group 2 (TAU 🡪 MBERT) | Analytical comparison |
| --- | --- | --- | --- |
| *Number of patients receiving psychotherapeutic treatment* | | | |
| TSST1 | *n* = 6 (CBT = 5, others = 1) | *n* = 12 (CBT = 8, PD = 3, others = 1) | *χ*^2^(1) = 1.702, *p* > .05, *V* = .206 |
| 🡪 |  | *n* = 2 stopped; *n* = 1 started a new one |  |
| TSST2 | *n* = 6 (CBT = 5, others = 1) | *n* = 11 (CBT = 8, PD = 2, others = 1) | *χ*^2^(1) = 1.018, *p* > .05, *V* = .160 |
| 🡪 | *n* = 1 stopped;  *n* = 1 started a new one | *n* = 1 stopped;  *n* = 2 started a new one^a^ |  |
| TSST3 | *n* = 6 (CBT = 5, others = 1) | *n* = 12 (CBT = 8, PD = 2, others = 2) | *χ*^2^(1) = 1.702, *p* > .05, *V* = .206 |
| *Number of patients receiving antidepressant medication* | | | |
| TSST1 | *n* = 7 | *n* = 10 | *χ*^2^(1) = 0.136, *p* > .05, *V* = 0.058 |
| 🡪 | *n* = 2 stopped | *n* = 1 stopped |  |
| TSST2 | *n* = 5 | *n* = 9 | *χ*^2^(1) = 0.583, *p* > .05, *V* = 0.121 |
| 🡪 | *n* = 1 started a new one | *n* = 1 stopped; *n* = 1 started a new one |  |
| TSST3 | *n* = 6 | *n* = 9 | *χ*^2^(1) = 0.167, *p* > .05, *V* = 0.065 |

**Full model specifications and R-Syntaxes of the linear mixed models**

*Model 1 (Growth model):*

Equation:

$$Y_{ij}= \beta_{0}+ \beta_{1}*StudyPhase_{ij}+ \beta_{2}*Time_{ij}+ \beta_{3}*\left( StudyPhase_{ij}\times Time_{ij} \right)+u_{0j}+u_{1j}*StudyPhase_{ij}+\varepsilon_{ij}$$

Where:

- $Y_{ij}$ = outcome variable for individual j at time point i
- $StudyPhase_{ij}$ = study phase (i.e. MBERT, TAU)
- $Time_{ij}$ = time (i.e. assessment point)
- $\beta_{0}$ = fixed intercept
- $\beta_{1}$, $\beta_{2}$, $\beta_{3}$ = fixed effects for Study Phase, Time, and their interaction
- $u_{0j}$ = random intercept for participant j
- $u_{1j}$ = random slope for Phase for participant j
- $\varepsilon_{ij}$ = residual error term

This model corresponds to the R code:

lmer(dependent variable ~ study phase * time + (1 + study phase | Subject), data, REML = F)

*Model 2 (Temporal dynamics of change):*

Equation:

$$Y_{t+1,j}=\beta_{0}+ \beta_{1}*Y_{t,j}+ \beta_{2}*X_{t,j}+ \beta_{3}*X_{t-1,j}+\beta_{4}*\left( StudyPhase_{t,j}\times X_{t,j} \right)+u_{0j}+\varepsilon_{t+1,j}$$

Where:

- $Y_{t+1,j}$ = outcome variable at time t+1 for participant j
- $Y_{t,j}$ = outcome variable at time t
- $X_{t,j}$ = predictor variable at time t
- $X_{t-1,j}$ = predictor variable at time t–1
- $StudyPhase_{t,j}$ = study phase at time t
- $\beta_{0}$ = fixed intercept
- $\beta_{1}$ to $\beta_{4}$ = fixed effects
- $u_{0j}$ = random intercept for participant j
- $\varepsilon_{t+1,j}$ = residual error at time t+1

This model corresponds to the R code:

lmer(outcome variable at t+1 ~ outcome variable at t + predictor variable at t * study phase + predictor variable at t-1 + (1 | Subject), data, REML = F)

**Supplementary Results**

**Factor analysis**

Table S4
*Factor loadings of the 15 items used in the assessment of rumination, self-efficacy, self-kindness and mindful distancing.*

| Item | Factor | | | | |
| --- | --- | --- | --- | --- | --- |
|  | 1 | 2 | 3 | 4 | 5 |
| self-efficacy 1 | **0.810** | 0.101 | -0.264 | -0.062 | 0.124 |
| self-efficacy 2 | **0.936** | -0.061 | 0.146 | 0.066 | 0.026 |
| self-efficacy 3 | **0.977** | 0.018 | 0.033 | 0.033 | 0.067 |
| rumination 1 | 0.022 | **1.047** | -0.003 | -0.041 | 0.016 |
| rumination 2 | -0.242 | **0.505** | 0.250 | 0.160 | -0.281 |
| rumination 3 | 0.022 | **0.791** | 0.156 | 0.033 | -0.165 |
| rumination 4 | 0.037 | 0.178 | **0.877** | 0.050 | -0.014 |
| rumination 5 | -0.250 | 0.215 | **0.565** | -0.040 | 0.052 |
| rumination 6 | -0.456 | 0.265 | 0.407 | -0.217 | 0.257 |
| self-kindness 1 | 0.040 | 0.208 | -0.366 | **0.681** | 0.142 |
| self-kindness 2 | 0.164 | -0.095 | 0.107 | **0.850** | 0.041 |
| self-kindness 3 | -0.075 | -0.037 | 0.007 | **0.795** | 0.284 |
| mindful distancing 1 | 0.076 | -0.028 | 0.009 | 0.046 | **0.903** |
| mindful distancing 2 | 0.072 | -0.027 | 0.016 | 0.100 | **0.853** |
| mindful distancing 3 | 0.021 | -0.105 | 0.003 | 0.158 | **0.725** |

*Notes.* Extraction method: Maximum likelihood; Rotation method: Oblimin. Bold numbers indicate loadings larger than 0.5. The analysis was based on *n* = 4925.

**Cronbach’s Alpha of the EMA questionnaire**

Table S5
*Cronbach’s Alpha [95 % confidence interval] statistics for the different scales of the EMA questionnaire. The values are based on each patients’ last EMA data entry. Note, however, that Cronbach’s alpha for other time points was comparable.*

| Self-efficacy | Rumination | Self-kindness | Mindful distancing |
| --- | --- | --- | --- |
| .97 [.95 - .99] | .90 [.85 - .95] | .84 [.74 - .93] | .88 [.81 - .94] |

**Changes within the dependent variables**

Table S6
*Results of the rmMANOVA for the factors group (Group 1 vs. Group 2) and time (baseline, halftime, endpoint) and post-hoc t-tests. Significance was determined using the Benjamini-Hochberg correction [12]. Both uncorrected (p) and corrected p-values (p_corr_) are reported. Boldface p-values indicate statistical significance.*

| **interaction effect of time and group:**  *Wilks λ* = 0.484, *F*(12,142) = 5.182, ***p* < .001**, *η_p_²* = 0.31 | | | |
| --- | --- | --- | --- |
| Rumination | *F*(2,76) = 6.196, ***p_corr_* = .038** (*p* = .003), *η_p_²* = 0.14 | | |
| Stress | *F*(2,76) = 0.478, *p_corr_* = .05 (*p* = .622), *η_p_²* = 0.01 | | |
| Self-efficacy | *F*(2,76) = 7.550, ***p_corr_* = .033** (*p* = .001), *η_p_²* = 0.17 | | |
| Self-kindness | *F*(2,76) = 32.065, ***p_corr_* = .025** (*p* < .001), *η_p_²* = 0.46 | | |
| Mindful distancing | *F*(2,76) = 16.089, ***p_corr_* = .029** (*p* < .001), *η_p_²* = 0.30 | | |
| Sleep quality | *F*(2,76) = 1.707, *p_corr_* = .046 (*p* = .188), *η_p_²* = 0.04 | | |
|  | | | |
| **main effect of time:**  *Wilks λ* = 0.232, *F*(12,142) = 12.747, ***p* < .001**, *η_p_²* = 0.52 | | | |
| Rumination | *F*(2,76) = 34.409, ***p_corr_* = .004** (*p* < .001), *η_p_²* = 0.48 | | |
| Stress | *F*(2,76) = 16.644, ***p_corr_* = .008** (*p* < .001), *η_p_²* = 0.31 | | |
| Self-efficacy | *F*(2,76) = 41.258, ***p_corr_* = .013** (*p* < .001), *η_p_²* = 0.52 | | |
| Self-kindness | *F*(2,76) = 95.831, ***p_corr_* = .017** (*p* <.001), *η_p_²* = 0.72 | | |
| Mindful distancing | *F*(2,76) = 75.694, ***p_corr_* = .021** (*p* < .001), *η_p_²* = 0.67 | | |
| Sleep quality | *F*(2,76) = 2.332, *p_corr_* = .042 (*p* = .104), *η_p_²* = 0.06 | | |
|  | | | |
| **post-hoc t-tests: between groups** | | | |
|  | baseline | halftime | endpoint |
| Rumination | *t*(38) = 1.138, *p_corr_* = .039  (*p* = .131), *d* = 0.36 | *t*(38) = -1.479, *p_corr_* = .033  (*p* = .074), *d* = 0.47 | *t*(38) = 2.211, ***p_corr_* = .028**  (*p* = .017), *d* = 0.70 |
| Stress | *t*(38) = 0.781, *p_corr_* = .044  (*p* = .220), *d* = 0.25 | *t*(38) = -0.076, *p_corr_* = .05  (*p* = .470), *d* = 0.02 | *t*(38) = .848, *p_corr_* = .042  (*p* = .201), *d* = 0.27 |
| Self-efficacy | *t*(38) = -0.646, *p_corr_* = .046  (*p* = .261), *d* = 0.20 | *t*(38) = 1.326, *p_corr_* = .035  (*p* = .096), *d* = 0.42 | *t*(38) = -2.206, ***p_corr_* = .029**  (*p* = .017), *d* = 0.71 |
| Self-kindness | *t*(38) = -0.279, *p_corr_* = .047  (*p* = .391), *d* = 0.09 | *t*(38) = 3.950, ***p_corr_* = .021**  (*p* < .001), *d* = 1.25 | *t*(38) = -2.220, ***p_corr_* = .025**  (*p* = .016), *d* = 0.70 |
| Mindful distancing | *t*(38) = 0.936, *p_corr_* = .040  (*p* = .177), *d* = 0.30 | *t*(38) = 3.303, ***p_corr_* = .022**  (*p* = .001), *d* = 1.05 | *t*(38) = -1.187, *p_corr_* = .038  (*p* = .121), *d* = 0.38 |
| Sleep quality | *t*(38) = -2.817, ***p_corr_* = .024**  (*p* = .004), *d* = 0.89 | *t*(38) = -1.213, *p_corr_* = .036  (*p* = .116), *d* = 0.38 | *t*(38) = -0.829, *p_corr_* = .043  (*p* = .206), *d* = 0.26 |
|  | |  |  |
| **post-hoc t-tests: between time points** | | | |
|  | baseline 🡪 halftime | halftime 🡪 endpoint | baseline 🡪 endpoint |
| Rumination | *t*(39) = -3.832, ***p_corr_* = .003** (*p* < .001), *d* = 0.61 | *t*(39) = -3.899, ***p_corr_* = .004** (*p* < .001), *d* = 0.62 | *t*(39) = -8.109, ***p_corr_* = .001** (*p* < .001), *d* = 1.28 |
| Stress | *t*(39) = -3.491, ***p_corr_* = .007** (*p* < .001), *d* = 0.55 | *t*(39) = -2.238, ***p_corr_* = .026** (*p* = .016), *d* = 0.35 | *t*(39) = -5.587, ***p_corr_* = .006** (*p* < .001), *d* = 0.88 |
| Self-efficacy | *t*(39) = 4.001, ***p_corr_* = .010** (*p* < .001), *d* = 0.63 | *t*(39) = 4.639, ***p_corr_* = .011** (*p* < .001), *d* = 0.73 | *t*(39) = 8.248, ***p_corr_* = .008** (*p* < .001), *d* = 1.30 |
| Self-kindness | *t*(39) = 4.572, ***p_corr_* = .014** (*p* < .001), *d* = 0.72 | *t*(39) = 5.078, ***p_corr_* = .015** (*p* < .001), *d* = 0.80 | *t*(39) = 12.668, ***p_corr_* = .013** (*p* < .001), *d* = 2.00 |
| Mindful distancing | *t*(39) = 4.313, ***p_corr_* = .018** (*p* < .001), *d* = 0.68 | *t*(39) = 5.847, ***p_corr_* = .019** (*p* < .001), *d* = 0.92 | *t*(39) = 11.029, ***p_corr_* = .017** (*p* < .001), *d* = 1.74 |
| Sleep quality | *t*(39) = 1.934, ***p_corr_* = .031** (*p* = .030), *d* = 0.31 | *t*(39) = 0.095, *p_corr_* = .049 (*p* = .463), *d* = 0.02 | *t*(39) = 1.633, *p_corr_* = .032 (*p* = .055), *d* = 0.26 |

**Model 1: Dependent variable ~ study phase * time**

As the variables rumination and stress were not normally distributed but right-skewed and therefore violated an important assumption of LMMs, we recalculated the models analyzing rumination or stress as dependent variables with log-transformed data. Note that as the scale for the subjective stress rating ranged from 0 to 100, we added 1 to each value before the log-transformation. However, results did not change when using the log-transformed variables (see Table S7).

Table S7
*Results of the Linear Mixed Models exploring the association between subjective stress / rumination and study phase and time, using log-transformed variables to correct for non-normally distributed data. AIC = Akaike Information Criterion; BIC = Bayesian-Information-Criterion; R^2^ = variance explained by the fixed effects.
^a^Coding of the Study Phase variable: 0 = TAU, 1 = MBERT. ^b^Note, that for each dependent variable the time predictor is the one with the best prediction (uncorrected for rumination, logarithmic time for stress). #p < .1, *p < .05, **p < .01, ***p < .001.*

| Dependent variables | Rumination | Stress |
| --- | --- | --- |
| Intercept | 0.707*** (0.044) | 3.198*** (0.140) |
| Study Phase^a^ | 0.103* (0.045) | -0.002 (0.097) |
| Time^b^ | -0.001* (0.000) | -0.006*** (0.001) |
| Study Phase^a^ * Time^b^ | -0.004*** (0.0004) | 0.001 (0.002) |
| AIC | 2622.6 | 14625.5 |
| BIC | 2674.6 | 14677.5 |
| R^2^ | 0.048 | 0.010 |

**Changes in the frequency of trigger occurance**

Table S8
*Results of the rmMANOVA for the factors group (Group 1 vs. Group 2) and time (phase 1, phase 2, phase 3) and relevant post-hoc t-tests. Significance was determined using the Benjamini-Hochberg correction [12]. Both uncorrected (p) and corrected p-values (p_corr_) are reported. Boldface p-values indicate statistical significance. In cases of violated sphericity, the Huynh-Feldt correction (HF) was used.*

| **interaction effect of time and group:**  *Wilks λ* = 0.676, *F*(14,84) = 1.298, *p* = .226, *η_p_²* = 0.18 | | | |
| --- | --- | --- | --- |
|  | | | |
| **main effect of time:**  *Wilks λ* = 0.478, *F*(14,84) = 2.683, ***p* = .003**, *η_p_*^2^ = 0.31 | | | |
| Trigger 1 | HF: *F*(1.519,36.462) = 9.936, ***p_corr_* = .003** (*p* < .001), *η_p_²* = 0.29 | | |
| Trigger 2 | *F*(2,48) = 1.021, *p_corr_* = .025 (*p* = .368), *η_p_²* = 0.04 | | |
| Trigger 3 | HF: *F*(1.550,37.195) = 1.321, *p_corr_* = .016 (*p* = .274), *η_p_²* = 0.05 | | |
| Trigger 4 | *F*(2,48) = 4.917, *p_corr_* = .006 (*p* = .011), *η_p_²* = 0.17 | | |
| Trigger 5 | *F*(2,48) = 3.754, *p_corr_* = .009 (*p* = .031), *η_p_²* = 0.14 | | |
| Trigger 6 | *F*(2,48) = 0.023, *p_corr_* = .050 (*p* = .977), *η_p_²* = 0.00 | | |
| Trigger 7 | *F*(2,48) = 0.149, *p_corr_* = .044 (*p* = .862), *η_p_²* = 0.01 | | |
| Trigger 8 | HF: *F*(1.458,34.982) = 0.225, *p_corr_* = .034 (*p* = .729), *η_p_²* = 0.01 | | |
|  | | | |
| **post-hoc t-tests: between time points** | | | |
|  | Phase 1 🡪 Phase 2 | Phase 2 🡪 Phase 3 | Phase 1 🡪 Phase 3 |
| Trigger 1 | *t*(25) = 2.736, ***p_corr_* = .033** (*p* = .006), *d* = 0.54 | *t*(25) = 1.793, *p_corr_* = .038 (*p* = .043), *d* = 0.35 | *t*(25) = 3.519, ***p_corr_* = .017** (*p* < .001), *d* = 0.69 |

**Changes in triggers effects on subjective stress and rumination**

Table S9
*Results of the rmMANOVA for the factors group (Group 1 vs. Group 2), time (phase 1, phase 2, phase 3) and trigger (trigger 1 to trigger 8) and relevant post-hoc t-tests. Significance was determined using the Benjamini-Hochberg correction [12]. Both uncorrected (p) and corrected p-values (p_corr_) are reported. Boldface p-values indicate statistical significance. In cases of violated sphericity, the Huynh-Feldt correction (HF) was used. For post-hoc comparisons between triggers see Table S10.*

| **Constant term:**  *Wilks λ* = 0.087, *F*(2,23) = 120.985, ***p* < .001**, *η_p_²* = 0.91 | | | |
| --- | --- | --- | --- |
|  | | | |
| **interaction effect of time, group and trigger:**  *Wilks λ* = 0.912, *F*(28,670) = 1.133, *p* = .291, *η_p_²* = 0.05 | | | |
|  | | | |
| **interaction effect of group and trigger:**  *Wilks λ* = 0.892, *F*(14,334) = 1.407, *p* = .148, *η_p_²* = 0.06 | | | |
|  | | | |
| **interaction effect of time and trigger:**  *Wilks λ* = 0.864, *F*(28,670) = 1.821, ***p* = .006**, *η_p_²* = 0.07 | | | |
| Stress | HF: *F*(11.464,25.139) = 2.395, ***p_corr_* = 0.031** (*p* = .007), *η_p_²* = 0.09 | | |
| Rumination | HF: *F*(11.302,271.241) = 1.531, *p_corr_* = .044 (*p* = 0.118), *η_p_²* = 0.06 | | |
|  | | | |
| **interaction effect of time and group:**  *Wilks λ* = 0.816, *F*(4,94) = 2.515, ***p* = .047**, *η_p_²* = 0.10 | | | |
| Stress | *F*(2,48) = 0.596, *p_corr_* = .050 (*p* = 0.551), *η_p_²* = 0.02 | | |
| Rumination | HF: *F*(1.763,42.318) = 4.540, ***p_corr_* = .038** (*p* = 0.020), *η_p_²* = 0.159 | | |
|  | | | |
| **main effect of time:**  *Wilks λ* = 0.368, *F*(4,94) = 15.250, ***p* < .001**, *η_p_²* = 0.39 | | | |
| Stress | *F*(2,48) = 21.547, ***p_corr_* = .013** (*p* < .001), *η_p_²* = 0.473 | | |
| Rumination | HF: *F*(1.763,42.318) = 40.806, ***p_corr_* = .006** (*p* < .001), *η_p_²* = 0.63 | | |
|  | | | |
| **main effect of trigger:**  *Wilks λ* = 0.363, *F*(14,334) = 15.767, ***p* < .001**, *η_p_²* = 0.40 | | | |
| Stress | HF: *F*(5.986,143.658) = 19.672, ***p_corr_* = .025** (*p* < .001), *η_p_²* = 0.450 | | |
| Rumination | HF: *F*(5.899,141.574) = 18.862, ***p_corr_* = .019** (*p* < .001), *η_p_²* = 0.44 | | |
|  | | | |
| **post-hoc t-tests: between groups** | | | |
| *Rumination* | | | |
|  | Phase 1 | Phase 2 | Phase 3 |
| Trigger 1 | *t*(24) = -0.320, *p_corr_* = .047 (*p* = .376), *d* = 0.13 | *t*(24) = -1.683, *p_corr_* = .027 (*p* = .053), *d* = 0.67 | *t*(24) = 0.217, *p_corr_* = .048  (*p* = .415), *d* = 0.09 |
| Trigger 2 | *t*(24) = 1.079, *p_corr_* = .035  (*p* = .146), *d* = 0.39 | *t*(24) = -0.921, *p_corr_* = .037  (*p* = .183), *d* = 0.37 | *t*(24) = 0.994, *p_corr_* = .037  (*p* = .167), *d* = 0.28 |
| Trigger 3 | *t*(24) = -1.129, *p_corr_* = .035  (*p* = .138), *d* = 0.48 | *t*(24) = -2.107, *p_corr_* = .023  (*p* = .023), *d* = 0.84 | *t*(24) = -0.085, *p_corr_* = .049  (*p* = .467), *d* = 0.04 |
| Trigger 4 | *t*(24) = 1.299, *p_corr_* = .033  (*p* = .103), *d* = 0.52 | *t*(24) = 0.370, *p_corr_* = .046  (*p* = .357), *d* = 0.15 | *t*(24) = 0.842, *p_corr_* = .038  (*p* = .204), *d* = 0.33 |
| Trigger 5 | *t*(24) = 0.022, *p_corr_* = .050  (*p* = .491), *d* = 0.01 | *t*(24) = -1.413, *p_corr_* = .031  (*p* = .085), *d* = 0.56 | *t*(24) = -0.371, *p_corr_* = .06  (*p* = .357), *d* = 0.15 |
| Trigger 6 | *t*(24) = -0.276, *p_corr_* = .047  (*p* = .392), *d* = 0.11 | *t*(24) = -0.666, *p_corr_* = .042  (*p* = .256), *d* = 0.26 | *t*(24) = 1.628, *p_corr_* = .027  (*p* = .058), *d* = 0.65 |
| Trigger 7 | *t*(24) = -2.460, ***p_corr_* = .021**  (*p* = .011), *d* = 0.91 | *t*(24) = -1.465, *p_corr_* = .030  (*p* = .080), *d* = 0.52 | *t*(24) = -0.821, *p_corr_* = .039  (*p* = .210), *d* = 0.33 |
| Trigger 8 | *t*(24) = -0.768, *p_corr_* = .040  (*p* = .225), *d* = 0.31 | *t*(24) = -1.409, *p_corr_* = .032  (*p* = .090), *d* = 0.48 | *t*(24) = -1.458, *p_corr_* = .031  (*p* = .083), *d* = 0.49 |
|  | | | |
| **post-hoc t-tests: between time points** | | | |
| *Stress* | | | |
|  | Phase 1 🡪 Phase 2 | Phase 2 🡪 Phase 3 | Phase 1 🡪 Phase 3 |
| Trigger 1 | *t*(25) = 0.186, *p_corr_* = .049  (*p* = .427), *d* = 0.04 | *t*(25) = 0.455, *p_corr_* = .045  (*p* = .326), *d* = 0.09 | *t*(25) = 0.673, *p_corr_* = .042  (*p* = .254), *d* = 0.13 |
| Trigger 2 | *t*(25) = 2.687, ***p_corr_* = .019**  (*p* = .006), *d* = 0.53 | *t*(25) = 1.068, *p_corr_* = .036  (*p* = .148), *d* = 0.21 | *t*(25) = 3.074, ***p_corr_* = .016**  (*p* = .003), *d* = 0.60 |
| Trigger 3 | *t*(25) = 1.949, *p_corr_* = .025  (*p* = .031), *d* = 0.38 | *t*(25) = 0.562, *p_corr_* = .044  (*p* = .290), *d* = 0.11 | *t*(25) = 2.163, ***p_corr_* = .022**  (*p* = .020), *d* = 0.42 |
| Trigger 4 | *t*(25) = 3.737, ***p_corr_* = .007**  (*p* < .001), *d* = 0.73 | *t*(25) = 2.233, ***p_corr_* = .022**  (*p* = .017), *d* = 0.44 | *t*(25) = 4.550, ***p_corr_* = .007**  (*p* < .001), *d* = 0.89 |
| Trigger 5 | *t*(25) = 0.498, *p_corr_* = .044  (*p* = .312), *d* = 0.10 | *t*(25) = 2.382, ***p_corr_* = .021**  (*p* = .013), *d* = 0.47 | *t*(25) = 2.858, ***p_corr_* = .017**  (*p* = .004), *d* = 0.56 |
| Trigger 6 | *t*(25) = 1.537, *p_corr_* = .029  (*p* = .068), *d* = 0.30 | *t*(25) = 1.067, *p_corr_* = .036  (*p* = .148), *d* = 0.21 | *t*(25) = 3.413, ***p_corr_* = .014**  (*p* = .001), *d* = 0.67 |
| Trigger 7 | *t*(25) = 0.820, *p_corr_* = .039  (*p* = .210), *d* = 0.16 | *t*(25) = 1.425, *p_corr_* = .031  (*p* = .083), *d* = 0.28 | *t*(25) = 2.896, ***p_corr_* = .017**  (*p* = .004), *d* = 0.57 |
| Trigger 8 | *t*(25) = 0.376, *p_corr_* = .045  (*p* = .355), *d* = 0.07 | *t*(25) = -1.436, *p_corr_* = .030  (*p* = .082), *d* = 0.28 | *t*(25) = 0.095, *p_corr_* = .049  (*p* = .463), *d* = 0.02 |
| *Rumination* | | | |
|  | Phase 1 🡪 Phase 2 | Phase 2 🡪 Phase 3 | Phase 1 🡪 Phase 3 |
| Trigger 1 | *t*(25) = 1.588, *p_corr_* = .028  (*p* = .062), *d* = 0.31 | *t*(25) = 1.977, *p_corr_* = .024  (*p* = .030), *d* = 0.39 | *t*(25) = 3.929, ***p_corr_* = .007**  (*p* < .001), *d* = 0.77 |
| Trigger 2 | *t*(25) = 3.443, ***p_corr_* = .014**  (*p* = .001), *d* = 0.68 | *t*(25) = 1.413, *p_corr_* = .031  (*p* = .085), *d* = 0.28 | *t*(25) = 4.739, ***p_corr_* = .007**  (*p* < .001), *d* = 0.93 |
| Trigger 3 | *t*(25) = 1.917, *p_corr_* = .025  (*p* = .033), *d* = 0.38 | *t*(25) = 1.449, *p_corr_* = .030  (*p* = .080), *d* = 0.28 | *t*(25) = 3.702, ***p_corr_* = .007**  (*p* < .001), *d* = 0.73 |
| Trigger 4 | *t*(25) = 2.376, ***p_corr_* = .021**  (*p* = .013), *d* = 0.47 | *t*(25) = 1.645, *p_corr_* = .027  (*p* = .056), *d* = 0.32 | *t*(25) = 3.493, ***p_corr_* = .007**  (*p* < .001), *d* = 0.69 |
| Trigger 5 | *t*(25) = 1.063, *p_corr_* = .036  (*p* = .149), *d* = 0.21 | *t*(25) = 3.239, ***p_corr_* = .015**  (*p* = .002), *d* = 0.64 | *t*(25) = 3.839, ***p_corr_* = .007**  (*p* < .001), *d* = 0.75 |
| Trigger 6 | *t*(25) = 2.594, ***p_corr_* = .020**  (*p* = .008), *d* = 0.51 | *t*(25) = 1.333, *p_corr_* = .032  (*p* = .097), *d* = 0.26 | *t*(25) = 4.614, ***p_corr_* = .007**  (*p* < .001), *d* = 0.91 |
| Trigger 7 | *t*(25) = 2.816, ***p_corr_* = .018**  (*p* = .005), *d* = 0.55 | *t*(25) = 1.248, *p_corr_* = .033  (*p* = .112), *d* = 0.25 | *t*(25) = 3.522, ***p_corr_* = .007**  (*p* < .001), *d* = 0.69 |
| Trigger 8 | *t*(25) = 0.793, *p_corr_* = .040  (*p* = .218), *d* = 0.16 | *t*(25) = 1.282, *p_corr_* = .033  (*p* = .106), *d* = 0.25 | *t*(25) = 1.178, *p_corr_* = .034  (*p* = .125), *d* = 0.23 |

Table S10

*Results of the post-hoc comparisons between triggers during the different phases. Significance was determined using the Benjamini-Hochberg correction [12]. Both uncorrected (p) and corrected p-values (pcorr) are reported. Boldface p-values indicate statistical significance.*

| ***Stress*** | | | | | | | |
| --- | --- | --- | --- | --- | --- | --- | --- |
| *Phase 1* | Trigger 2 | Trigger 3 | Trigger 4 | Trigger 5 | Trigger 6 | Trigger 7 | Trigger 8 |
| Trigger 1 | *t*(25) = -5.184,  ***p_corr_* = .007** (*p* < .001), *d* = 1.02 | *t*(25) = -6.568,  ***p_corr_* = .007** (*p* < .001), *d* = 1.29 | *t*(25) = -5.998,  ***p_corr_* = .007** (*p* < .001), *d* = 1.18 | *t*(25) = -4.076,  ***p_corr_* = .007** (*p* < .001), *d* = 0.80 | *t*(25) = -7.682,  ***p_corr_* = .007** (*p* < .001), *d* = 1.51 | *t*(25) = -0.761,  *p_corr_* = .041 (*p* = .227), *d* = 0.15 | *t*(25) = 1.754,  *p_corr_* = .026 (*p* = .046), *d* = 0.34 |
| Trigger 2 |  | *t*(25) = 0.411,  *p_corr_* = .045 (*p* = .342), *d* = 0.08 | *t*(25) = -1.092,  *p_corr_* = .035 (*p* = .143), *d* = 0.21 | *t*(25) = 1.357,  *p_corr_* = .032 (*p* = .093), *d* = 0.27 | *t*(25) = -0.323,  *p_corr_* = .047 (*p* = .375), *d* = 0.06 | *t*(25) = 4.474,  ***p_corr_* = .007** (*p* < .001), *d* = 0.88 | *t*(25) = 7.297,  ***p_corr_* = .007** (*p* < .001), *d* = 1.43 |
| Trigger 3 |  |  | *t*(25) = -1.566,  *p_corr_* = .028 (*p* = .065), *d* = 0.31 | *t*(25) = 0.801,  *p_corr_* = .040 (*p* = .215), *d* = 0.16 | *t*(25) = -1.001,  *p_corr_* = .036 (*p* = .163), *d* = 0.20 | *t*(25) = 3.929,  ***p_corr_* = .007** (*p* < .001), *d* = 0.77 | *t*(25) = 6.615,  ***p_corr_* = .007** (*p* < .001), *d* = 1.30 |
| Trigger 4 |  |  |  | *t*(25) = 2.820,  ***p_corr_* = .018** (*p* = .005), *d* = 0.55 | *t*(25) = 0.840,  *p_corr_* = .038 (*p* = .204), *d* = 0.17 | *t*(25) = 6.111,  ***p_corr_* = .007** (*p* < .001), *d* = 1.20 | *t*(25) = 7.553,  ***p_corr_* = .007** (*p* < .001), *d* = 1.48 |
| Trigger 5 |  |  |  |  | *t*(25) = -2.510,  ***p_corr_* = .020** (*p* = .009), *d* = 0.49 | *t*(25) = 4.008,  ***p_corr_* = .007** (*p* < .001), *d* = 0.79 | *t*(25) = 6.056,  ***p_corr_* = .007** (*p* < .001), *d* = 1.19 |
| Trigger 6 |  |  |  |  |  | *t*(25) = 5.291,  ***p_corr_* = .007** (*p* < .001), *d* = 1.04 | *t*(25) = 9.535,  ***p_corr_* = .007** (*p* < .001), *d* = 1.87 |
| Trigger 7 |  |  |  |  |  |  | *t*(25) = 2.736,  ***p_corr_* = .019** (*p* = .006), *d* = 0.54 |
| *Phase 2* | Trigger 2 | Trigger 3 | Trigger 4 | Trigger 5 | Trigger 6 | Trigger 7 | Trigger 8 |
| Trigger 1 | *t*(25) = -2.408,  ***p_corr_* = .021** (*p* = .012), *d* = 0.47 | *t*(25) = -2.997,  ***p_corr_* = .016** (*p* = .003), *d* = 0.59 | *t*(25) = -3.207,  ***p_corr_* = .015** (*p* = .002), *d* = 0.63 | *t*(25) = -3-393,  ***p_corr_* = .014** (*p* = .001), *d* = 0.67 | *t*(25) = -4.277,  ***p_corr_* = .007** (*p* < .001), *d* = 0.84 | *t*(25) = -0.009,  *p_corr_* = .005 (*p* = .496), *d* = 0.00 | *t*(25) = 1.929,  *p_corr_* = .025 (*p* = .033), *d* = 0.38 |
| Trigger 2 |  | *t*(25) = -0.678,  *p_corr_* = .004 (*p* = .252), *d* = 0.13 | *t*(25) = -1.191,  *p_corr_* = .034 (*p* = .123), *d* = 0.23 | *t*(25) = -0.864,  *p_corr_* = .038 (*p* = .198), *d* = 0.17 | *t*(25) = -1.303,  *p_corr_* = .033 (*p* = .102), *d* = 0.26 | *t*(25) = 2.120,  ***p_corr_* = .023** (*p* = .022), *d* = 0.42 | *t*(25) = 4.026,  ***p_corr_* = .007** (*p* < .001), *d* = 0.79 |
| Trigger 3 |  |  | *t*(25) = -0.574,  *p_corr_* = .043 (*p* = .285), *d* = 0.11 | *t*(25) = -0.379,  *p_corr_* = .045 (*p* = .354), *d* = 0.07 | *t*(25) = -0.892,  *p_corr_* = .037 (*p* = .190), *d* = 0.18 | *t*(25) = 2.940,  ***p_corr_* = .016** (*p* = .003), *d* = 0.58 | *t*(25) = 5.284,  ***p_corr_* = .007** (*p* < .001), *d* = 1.04 |
| Trigger 4 |  |  |  | *t*(25) = 0.184,  *p_corr_* = .049 (*p* = .428), *d* = 0.04 | *t*(25) = -0.477,  *p_corr_* = .044 (*p* = .319), *d* = 0.09 | *t*(25) = 2.850,  ***p_corr_* = .017** (*p* = .004), *d* = 0.56 | *t*(25) = 5.276,  ***p_corr_* = .007** (*p* < .001), *d* = 1.04 |
| Trigger 5 |  |  |  |  | *t*(25) = -0.573,  *p_corr_* = .043 (*p* = .286), *d* = 0.11 | *t*(25) = 2.664,  ***p_corr_* = .019** (*p* = .007), *d* = .52 | *t*(25) = 5.210,  ***p_corr_* = .007** (*p* < .001), *d* = 1.02 |
| Trigger 6 |  |  |  |  |  | *t*(25) = 3.910,  ***p_corr_* = .007** (*p* < .001), *d* = 0.77 | *t*(25) = 6.291,  ***p_corr_* = .007** (*p* < .001), *d* = 1.23 |
| Trigger 7 |  |  |  |  |  |  | *t*(25) = 1.838,  *p_corr_* = .026 (*p* = .039), *d* = 0.36 |
|  |  |  |  |  |  |  |  |
| *Phase 3* | Trigger 2 | Trigger 3 | Trigger 4 | Trigger 5 | Trigger 6 | Trigger 7 | Trigger 8 |
| Trigger 1 | *t*(25) = -1.924,  *p_corr_* = .025 (*p* = .033), *d* = 0.38 | *t*(25) = -2.489,  ***p_corr_* = .021** (*p* = .010), *d* = 0.49 | *t*(25) = -2.028,  *p_corr_* = .024 (*p* = .027), *d* = 0.40 | *t*(25) = -1.499,  *p_corr_* = .029 (*p* = .073), *d* = 0.29 | *t*(25) = -3.818,  ***p_corr_* = .007** (*p* < .001), *d* = 0.75 | *t*(25) = 1.185,  *p_corr_* = .034 (*p* = .124), *d* = 0.23 | *t*(25) = 1.461,  *p_corr_* = .030 (*p* = .078), *d* = 0.29 |
| Trigger 2 |  | *t*(25) = -0.885,  *p_corr_* = .038 (*p* = .192), *d* = 0.17 | *t*(25) = 0.232,  *p_corr_* = .048 (*p* = .409), *d* = 0.05 | *t*(25) = 0.786,  *p_corr_* = .040 (*p* = .220), *d* = 0.15 | *t*(25) = -1.950,  *p_corr_* = .025 (*p* = .031), *d* = 0.38 | *t*(25) = 2.861,  ***p_corr_* = .017** (*p* = .004), *d* = 0.56 | *t*(25) = 3.093,  ***p_corr_* = .015** (*p* = .002), *d* = 0.61 |
| Trigger 3 |  |  | *t*(25) = 1.003,  *p_corr_* = .036 (*p* = .163), *d* = 0.20 | *t*(25) = 1.558,  *p_corr_* = .029 (*p* = .066), *d* = 0.31 | *t*(25) = -0.668,  *p_corr_* = .042 (*p* = .255), *d* = 0.13 | *t*(25) = 3.991,  ***p_corr_* = .007** (*p* < .001), *d* = 0.78 | *t*(25) = 4.046,  ***p_corr_* = .007** (*p* < .001), *d* = 0.79 |
| Trigger 4 |  |  |  | *t*(25) = 0.625,  *p_corr_* = .043 (*p* = .269), *d* = 0.12 | *t*(25) = -2.023,  *p_corr_* = .024 (*p* = .027), *d* = 0.40 | *t*(25) = 2.608,  ***p_corr_* = .020** (*p* = .008), *d* = 0.51 | *t*(25) = 2.716,  ***p_corr_* = .019** (*p* = .006), *d* = 0.53 |
| Trigger 5 |  |  |  |  | *t*(25) = -2.480,  ***p_corr_* = .021** (*p* = .010), *d* = 0.49 | *t*(25) = 2.108,  *p_corr_* = .023 (*p* = .023), *d* = 0.41 | *t*(25) = 2.214,  ***p_corr_* = .022** (*p* = .018), *d* = 0.43 |
| Trigger 6 |  |  |  |  |  | *t*(25) = 4.423,  ***p_corr_* = .007** (*p* < .001), *d* = 0.87 | *t*(25) = 4.932,  ***p_corr_* = .007** (*p* < .001), *d* = 0.97 |
| Trigger 7 |  |  |  |  |  |  | *t*(25) = 0.764,  *p_corr_* = .040 (*p* = .226), *d* = 0.15 |
|  | | | | | | | |
| ***Rumination*** | | | | | | | |
| *Phase 1* | Trigger 2 | Trigger 3 | Trigger 4 | Trigger 5 | Trigger 6 | Trigger 7 | Trigger 8 |
| Trigger 1 | *t*(25) = -2.296,  ***p_corr_* = .022** (*p* = .015), *d* = 0.45 | *t*(25) = -1.562,  *p_corr_* = .028 (*p* = .065), *d* = 0.31 | *t*(25) = -1.666,  *p_corr_* = .027 (*p* = .054), *d* = 0.33 | *t*(25) = -0.419,  *p_corr_* = .045 (*p* = .339), *d* = 0.08 | *t*(25) = -3.782,  ***p_corr_* = .007** (*p* < .001), *d* = 0.74 | *t*(25) = 1.513,  *p_corr_* = .029 (*p* = .071), *d* = 0.30 | *t*(25) = 5.795,  ***p_corr_* = .007** (*p* < .001), *d* = 1.14 |
| Trigger 2 |  | *t*(25) = 0.649,  *p_corr_* = .043 (*p* = .261), *d* = 0.13 | *t*(25) = 0.327,  *p_corr_* = .046 (*p* = .373), *d* = 0.06 | *t*(25) = 1.786,  *p_corr_* = .026 (*p* = .043), *d* = 0.35 | *t*(25) = -1.176,  *p_corr_* = .034 (*p* = .125), *d* = 0.23 | *t*(25) = 3.304,  ***p_corr_* = .014** (*p* = .001), *d* = 0.65 | *t*(25) = 7.189,  ***p_corr_* = .007** (*p* < .001), *d* = 1.41 |
| Trigger 3 |  |  | *t*(25) = -0.322,  *p_corr_* = .047 (*p* = .375), *d* = 0.06 | *t*(25) = 0.818,  *p_corr_* = .039 (*p* = .211), *d* = 0.16 | *t*(25) = -2.039,  *p_corr_* = .023 (*p* = .026), *d* = 0.40 | *t*(25) = 2.561,  ***p_corr_* = .020** (*p* = .008), *d* = 0.50 | *t*(25) = 5.411,  ***p_corr_* = .007** (*p* < .001), *d* = 1.06 |
| Trigger 4 |  |  |  | *t*(25) = 1.331,  *p_corr_* = .032 (*p* = .098), *d* = 0.26 | *t*(25) = -1.481,  *p_corr_* = .029 (*p* = .076), *d* = 0.29 | *t*(25) = 2.895,  ***p_corr_* = .017** (*p* = .004), *d* = 0.57 | *t*(25) = 5.869,  ***p_corr_* = .007** (*p* < .001), *d* = 1.15 |
| Trigger 5 |  |  |  |  | *t*(25) = -3.540,  ***p_corr_* = .007** (*p* < .001), *d* = 0.69 | *t*(25) = 2.643,  ***p_corr_* = .019** (*p* = .007), *d* = 0.52 | *t*(25) = 5.937,  ***p_corr_* = .007** (*p* < .001), *d* = 1.16 |
| Trigger 6 |  |  |  |  |  | *t*(25) = 5.498,  ***p_corr_* = .007** (*p* < .001), *d* = 1.08 | *t*(25) = 9.548,  ***p_corr_* = .007** (*p* < .001), *d* = 1.87 |
| Trigger 7 |  |  |  |  |  |  | *t*(25) = 3.422,  ***p_corr_* = .014** (*p* = .001), *d* = 0.67 |
|  |  |  |  |  |  |  |  |
| *Phase 2* | Trigger 2 | Trigger 3 | Trigger 4 | Trigger 5 | Trigger 6 | Trigger 7 | Trigger 8 |
| Trigger 1 | *t*(25) = 0.373,  *p_corr_* = .046 (*p* = .356), *d* = 0.07 | *t*(25) = -0.463,  *p_corr_* = .044 (*p* = .324), *d* = 0.09 | *t*(25) = -0.646,  *p_corr_* = .043 (*p* = .262), *d* = 0.13 | *t*(25) = -0.324,  *p_corr_* = .046 (*p* = .374), *d* = 0.06 | *t*(25) = -1.705,  *p_corr_* = .026 (*p* = .050), *d* = 0.33 | *t*(25) = 3.793,  ***p_corr_* = .007** (*p* < .001), *d* = 0.74 | *t*(25) = 5.343,  ***p_corr_* = .007** (*p* < .001), *d* = 1.05 |
| Trigger 2 |  | *t*(25) = -0.954,  *p_corr_* = .037 (*p* = .175), *d* = 0.19 | *t*(25) = -1.205,  *p_corr_* = .034 (*p* = .120), *d* = 0.24 | *t*(25) = -0.711,  *p_corr_* = .041 (*p* = .242), *d* = 0.14 | *t*(25) = -1.438,  *p_corr_* = .030 (*p* = .081), *d* = 0.28 | *t*(25) = 2.534,  ***p_corr_* = .020** (*p* = .009), *d* = 0.50 | *t*(25) = .103,  ***p_corr_* = .007** (*p* < .001), *d* = 0.81 |
| Trigger 3 |  |  | *t*(25) = -0.278,  *p_corr_* = .047 (*p* = .392), *d* = 0.06 | *t*(25) = 0.189,  *p_corr_* = .048 (*p* = .426), *d* = 0.04 | *t*(25) = -0.849,  *p_corr_* = .038 (*p* = .202), *d* = 0.17 | *t*(25) = 3.987,  ***p_corr_* = .007** (*p* < .001), *d* = 0.78 | *t*(25) = 5.266,  ***p_corr_* = .007** (*p* < .001), *d* = 1.03 |
| Trigger 4 |  |  |  | *t*(25) = 0.531,  *p_corr_* = .044 (*p* = .300), *d* = 0.10 | *t*(25) = -0.693,  *p_corr_* = .041 (*p* = .247), *d* = 0.14 | *t*(25) = 3.541,  ***p_corr_* = .007** (*p* < .001), *d* = 0.69 | *t*(25) = 5.612,  ***p_corr_* = .007** (*p* < .001), *d* = 1.10 |
| Trigger 5 |  |  |  |  | *t*(25) = -1.227,  *p_corr_* = .034 (*p* = .116), *d* = 0.24 | *t*(25) = 3.360,  ***p_corr_* = .014** (*p* = .001), *d* = 0.66 | *t*(25) = 5.717,  ***p_corr_* = .007** (*p* < .001), *d* = 1.12 |
| Trigger 6 |  |  |  |  |  | *t*(25) = .665,  ***p_corr_* = .007** (*p* < .001), *d* = 0.92 | *t*(25) = 7.217,  ***p_corr_* = .007** (*p* < .001), *d* = 1.42 |
| Trigger 7 |  |  |  |  |  |  | *t*(25) = 1.611,  *p_corr_* = .028 (*p* = .060), *d* = 0.32 |
|  |  |  |  |  |  |  |  |
| *Phase 3* | Trigger 2 | Trigger 3 | Trigger 4 | Trigger 5 | Trigger 6 | Trigger 7 | Trigger 8 |
| Trigger 1 | *t*(25) = 0.664,  *p_corr_* = .042 (*p* = .256), *d* = 0.13 | *t*(25) = -0.157,  *p_corr_* = .049 (*p* = .438), *d* = 0.03 | *t*(25) = 0.052,  *p_corr_* = .050 (*p* = .479), *d* = 0.01 | *t*(25) = 2.003,  *p_corr_* = .024 (*p* = .028), *d* = 0.39 | *t*(25) = -1.230,  *p_corr_* = .033 (*p* = .115), *d* = 0.24 | *t*(25) = 5.405,  ***p_corr_* = .007** (*p* < .001), *d* = 1.06 | *t*(25) = 6.686,  ***p_corr_* = .007** (*p* < .001), *d* = 1.31 |
| Trigger 2 |  | *t*(25) = -0.815,  *p_corr_* = .039 (*p* = .211), *d* = 0.16 | *t*(25) = -0.710,  *p_corr_* = .041 (*p* = .242), *d* = 0.14 | *t*(25) = 0.857,  *p_corr_* = .038 (*p* = .200), *d* = 0.17 | *t*(25) = -1.989,  *p_corr_* = .024 (*p* = .029), *d* = 0.39 | *t*(25) = 2.974,  ***p_corr_* = .016** (*p* = .003), *d* = 0.58 | *t*(25) = 3.511,  ***p_corr_* = .007** (*p* < .001), *d* = 0.69 |
| Trigger 3 |  |  | *t*(25) = 0.204,  *p_corr_* = .048 (*p* = .420), *d* = 0.04 | *t*(25) = 2.017,  *p_corr_* = .024 (*p* = .027), *d* = 0.40 | *t*(25) = -1.151,  *p_corr_* = .035 (*p* = .130), *d* = 0.23 | *t*(25) = 4.890,  ***p_corr_* = .007** (*p* < .001), *d* = 0.96 | *t*(25) = 5.525,  ***p_corr_* = .007** (*p* < .001), *d* = 1.08 |
| Trigger 4 |  |  |  | *t*(25) = 1.874,  *p_corr_* = .026 (*p* = .036), *d* = 0.37 | *t*(25) = -1.573,  *p_corr_* = .028 (*p* = .064), *d* = 0.31 | *t*(25) = 3.687,  ***p_corr_* = .007** (*p* < .001), *d* = 0.72 | *t*(25) = 4.274,  ***p_corr_* = .007** (*p* < .001), *d* = 0.84 |
| Trigger 5 |  |  |  |  | *t*(25) = -3.263,  ***p_corr_* = .015** (*p* = .002), *d* = 0.64 | *t*(25) = 2.779,  ***p_corr_* = .018** (*p* = .005), *d* = 0.55 | *t*(25) = 3.450,  ***p_corr_* = .014** (*p* = .001), *d* = 0.68 |
| Trigger 6 |  |  |  |  |  | *t*(25) = 4.973,  ***p_corr_* = .007** (*p* < .001), *d* = 0.98 | *t*(25) = 6.382,  ***p_corr_* = .007** (*p* < .001), *d* = 1.25 |
| Trigger 7 |  |  |  |  |  |  | *t*(25) = 1.429,  *p_corr_* = .031 (*p* = .083), *d* = 0.28 |

Post-hoc *t*-tests of the performed rmMANOVA (see main article and Table S9) revealed no significant group differences with the exception of trigger 7 (sleep quality) with higher rumination after reporting sleep quality as stress-evoking event during phase 1 in group 2 (receiving MBERT after TAU) compared to group 1 (receiving MBERT before TAU). With respect to time, we found social interactions to elicit less subjective stress and rumination in phase 3 and phase 2 compared to phase 1. Additionally, work, internal causes and sleep quality elicited less subjective stress and rumination at the end (phase 3) compared to the beginning (phase 1). However, internal causes and bad sleep quality both already led to less rumination during phase 2 compared to phase 1. Patients were less stressed and reported less rumination after daily hassles during phase 3 compared to both phase 1 and phase 2. Finally, private obligations led to less subjective stress and rumination from phase 1 to phase 2 and from phase 1 to phase 3, and subjective stress was also lower after private obligations during phase 3 compared to phase 2. Interestingly, patients also reported less rumination without any stress-eliciting events (trigger 1: no answer) during phase 3 compared to phase 1. For a detailed presentation of the statistics see Table S9 and for a visualization of these results see Figure S1.

When comparing the triggers in terms of their impact on stress and rumination during phase 1, political events led to less subjective stress and rumination than all other triggers, and even to less rumination than without any reported trigger. Similarly, sleep quality reported as stressful event led to less stress and rumination than all other triggers (with the exception of trigger 8 (political events) and trigger 1 (no trigger)). However, internal causes elicited more subjective stress and rumination than daily hassles and no reported triggers. Also, private obligations elicited more stress than daily hassles. Further, social interactions, work, private obligations and daily hassles all led to more subjective stress than when no trigger was reported. Additionally, social interactions elicited more rumination as without the report of a stress-evoking trigger. Results were very similar during phase 2; however, political events did no longer lead to less stress nor rumination than bad sleep quality. Further, internal causes didn’t elicit more rumination than daily hassles or no reported trigger and also not higher stress than daily hassles, anymore. Also, private obligations did not elicit more stress than daily hassles. Surprisingly, patients reported more rumination after no reported triggers than after bad sleep quality during phase 2. During phase 3, results were comparable to phase 2, but sleep quality and daily hassles did no longer differ in their impact on subjective stress anymore, and reporting no stressful event no longer went along with less subjective stress than social interactions, private obligations and daily hassles. However, as during phase 1, internal causes again elicited more stress and rumination than daily hassles. For detailed statistics of these post-hoc comparisons see Table S9. Means and standard deviations of reported stress and rumination in dependence of each trigger and during each phase can be found in Table S11 as well as in Figure S1.

Taken together, internal causes and social interactions appear to play a major role in eliciting both subjective stress and rumination. Further, private obligations were highly stress-eliciting at the beginning of the study. However, in accordance with a reduction of both subjective stress and rumination throughout the study participation (see main article), also the impact of the triggers seems to diminish over time. Note that results concerning sleep quality and political events need to be interpreted with caution as these triggers were reported very scarcely and accounted for less than 5 % of all reported triggers during each phase.

Table S11
*Means (standard deviations) of subjectively perceived stress and rumination reported following the different triggers, separated by phase, respectively. The range for the stress rating was from 0-100 %, the range for the rumination rating was a Likert-scale ranging from 1-5.*

| Trigger | Phase 1 | | Phase 2 | | Phase 3 | |
| --- | --- | --- | --- | --- | --- | --- |
|  | Stress | Rumination | Stress | Rumination | Stress | Rumination |
| Trigger 1:  No answer | 10.538 (10.996) | 1.764 (1.032) | 10.235 (13.693) | 1.492 (0.955) | 9.632 (12.411) | 1.247 (0.722) |
| Trigger 2:  Social interaction | 42.519 (27.586) | 2.440 (1.323) | 24.740 (27.701) | 1.367 (1.466) | 20.320 (27.570) | 1.051 (1.341) |
| Trigger 3:  Work | 39.310 (21.626) | 2.208 (1.086) | 28.122 (22.809) | 1.636 (1.283) | 25.156 (25.443) | 1.290 (1.042) |
| Trigger 4:  Private obligations | 50.360 (29.334) | 2.321 (1.306) | 31.638 (28.081) | 1.720 (1.302) | 19.244 (21.966) | 1.234 (1.228) |
| Trigger 5:  Daily hassles | 33.555 (23.473) | 1.899 (1.304) | 30.576 (24.485) | 1.588 (1.127) | 16.702 (23.371) | 0.843 (0.980) |
| Trigger 6:  Internal causes | 44.766 (19.098) | 2.797 (1.011) | 34.546 (25.392) | 1.939 (1.175) | 29.180 (24.996) | 1.573 (1.079) |
| Trigger 7:  Sleep quality | 14.538 (19.527) | 1.183 (1.495) | 10.288 (23.923) | 0.463 (1.061) | 3.875 (13.509) | 0.212 (0.520) |
| Trigger 8:  Political events | 4.109 (12.008) | 0.349 (1.030) | 2.810 (11.940) | 0.158 (0.580) | 3.742 (14.653) | 0.092 (0.329) |


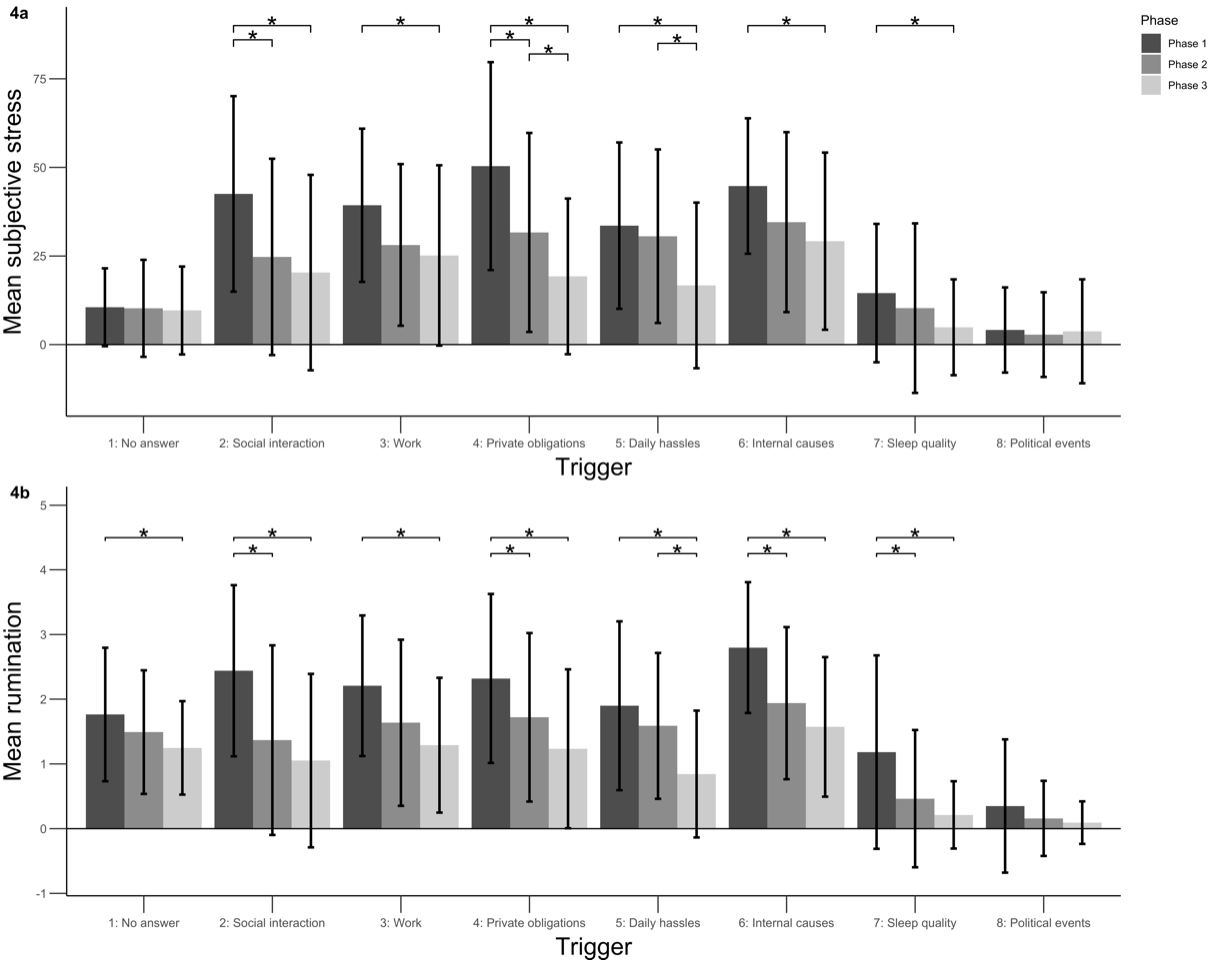


Figure S1. Means of subjective stress and rumination dependent of the type of trigger and study phase, reported via EMA in the course of the study participation. Note that significant differences between the different triggers within each phase are not displayed for reasons of easier data visualization.

**Supplementary Literature**

1. Mulligan, K. and K.R. Scherer, *Toward a working definition of emotion.* Emotion Review, 2012. **4**(4): p. 345-357.

2. Gross, J.J., *The emerging field of emotion regulation: An integrative review.* Review of general psychology, 1998. **2**(3): p. 271-299.

3. Rosenbaum, D., et al., *Amplitude of low frequency fluctuations (ALFF) of spontaneous and induced rumination in major depression: An fNIRS study.* Scientific reports, 2020. **10**(1): p. 21520.

4. Christoff, K., et al., *Mind-wandering as spontaneous thought: a dynamic framework.* Nature reviews neuroscience, 2016. **17**(11): p. 718-731.

5. Linehan, M., *DBT? Skills training manual*. 2014: Guilford Publications.

6. Schwarzer, R. and M. Jerusalem, *Generalized self-efficacy scale.* J. Weinman, S. Wright, & M. Johnston, Measures in health psychology: A user’s portfolio. Causal and control beliefs, 1995. **35**(37): p. 82-003.

7. Szkodny, L.E. and M.G. Newman, *Delineating Characteristics of Maladaptive Repetitive Thought: Development and Preliminary Validation of the Perseverative Cognitions Questionnaire.* Assessment, 2019. **26**(6): p. 1084-1104.

8. Nolen-Hoeksema, S. and J. Morrow, *A prospective study of depression and posttraumatic stress symptoms after a natural disaster: the 1989 Loma Prieta Earthquake.* Journal of Personality and Social Psychology, 1991. **61**(1): p. 115-121.

9. Hupfeld, J. and N. Ruffieux, *Validierung einer deutschen version der Self-Compassion Scale (SCS-D).* Zeitschrift fur Klinische Psychologie und Psychotherapie, 2011. **40**(2): p. 115-123.

10. Hautzinger, M., et al., *Beck depressions-Inventar: BDI II; manual*. 2009: Pearson Assessment.

11. Jerusalem, M. and R. Schwarzer, *SWE - Skala zur Allgemeinen Selbstwirksamkeitserwartung.* 2003.

12. Benjamini, Y. and Y. Hochberg, *Controlling the false discovery rate: a practical and powerful approach to multiple testing.* Journal of the Royal statistical society: series B (Methodological), 1995. **57**(1): p. 289-300.
